# Matrin3 regulates mitotic spindle dynamics by controlling alternative splicing of CDC14B

**DOI:** 10.1016/j.celrep.2023.112260

**Published:** 2023-03-15

**Authors:** Bruna R. Muys, Roshan L. Shrestha, Dimitrios G. Anastasakis, Lorinc Pongor, Xiao Ling Li, Ioannis Grammatikakis, Ahsan Polash, Raj Chari, Myriam Gorospe, Curtis C. Harris, Mirit I. Aladjem, Munira A. Basrai, Markus Hafner, Ashish Lal

**Affiliations:** 1 Regulatory RNAs and Cancer Section, Genetics Branch, Center for Cancer Research (CCR), National Cancer Institute (NCI), Bethesda, MD 20892, USA; 2 Genetics Branch, CCR, NCI, Bethesda, MD 20892, USA; 3 RNA Molecular Biology Laboratory, National Institute for Arthritis and Musculoskeletal and Skin Disease, Bethesda, MD 20892, USA; 4 Developmental Therapeutics Branch, CCR, NCI, NIH, Bethesda, MD 20892, USA; 5 Genome Modification Core, Frederick National Lab for Cancer Research, Frederick, MD 21701, USA; 6 Laboratory of Genetics and Genomics, National Institute on Aging, Baltimore, MD 21224, USA; 7 Laboratory of Human Carcinogenesis, CCR, NCI, Bethesda, MD 20892, USA; 8 Lead contact

## Abstract

Matrin3 is an RNA-binding protein that regulates diverse RNA-related processes, including mRNA splicing. Although Matrin3 has been intensively studied in neurodegenerative diseases, its function in cancer remains unclear. Here, we report Matrin3-mediated regulation of mitotic spindle dynamics in colorectal cancer (CRC) cells. We comprehensively identified RNAs bound and regulated by Matrin3 in CRC cells and focused on *CDC14B*, one of the top Matrin3 targets. Matrin3 knockdown results in increased inclusion of an exon containing a premature termination codon in the *CDC14B* transcript and simultaneous down-regulation of the standard *CDC14B* transcript. Knockdown of *CDC14B* phenocopies the defects in mitotic spindle dynamics upon Matrin3 knockdown, and the elongated and misoriented mitotic spindle observed upon Matrin3 knockdown are rescued upon overexpression of CDC14B, suggesting that *CDC14B* is a key downstream effector of Matrin3. Collectively, these data reveal a role for the *Matrin3/CDC14B* axis in control of mitotic spindle dynamics.

## In brief

Muys et al. find that the RNA-binding protein Matrin3—best characterized for its role in neurodegenerative diseases—affects cancer cell proliferation and mitotic spindle dynamics by binding and promoting proper processing of the mRNA encoding CDC14B, a conserved, key regulator of eukaryotic mitotic spindle formation.

## Graphical abstract

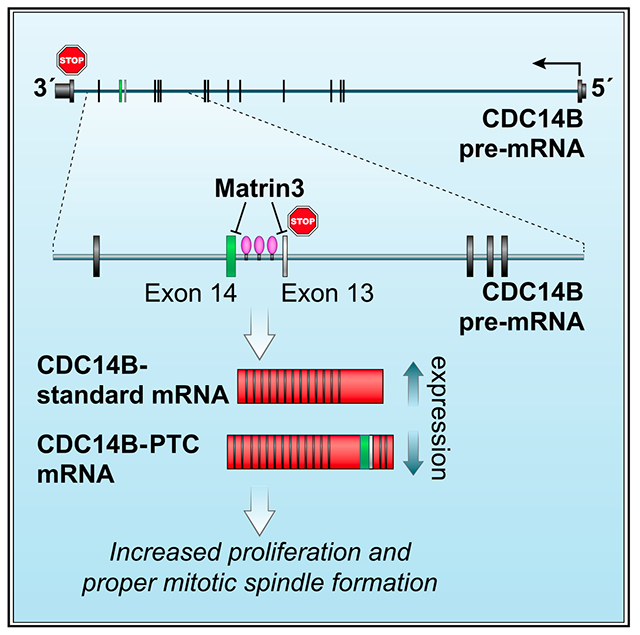

## INTRODUCTION

Matrin3 (MATR3) is a nucleic acid-binding protein^1,2^ that is conserved in vertebrates and is a major component of the nuclear matrix, a highly structured residual framework composed of lamins and ribonucleoproteins (RNPs).^3,4^ *In vitro*, Matrin3 can bind to DNA with its two zinc finger (ZF) motifs and to RNA through two tandem RNA recognition motifs (RRMs).^5^ By binding to DNA, Matrin3 can attach to specific chromatin structural elements termed matrix or scaffold attachment region (MAR or SAR, respectively).^6^ Consistently, Matrin3 can regulate chromatin organization.^7,8^ Nevertheless, most studies have focused on Matrin3 as an RNA-binding protein (RBP) regulating diverse—not necessarily related — RNA processes, including transcription,^9,10^ splicing,^11–13^ RNA stability,^14^ nuclear export,^15^ and nuclear retention of hyper-edited RNAs.^16^ Moreover, we previously reported that after DNA damage in colorectal cancer (CRC) cells, the long non-coding RNA (lncRNA) *PINCR* directly binds and recruits Matrin3 to enhancers of p53 target genes that depend on *PINCR* for p53-mediated induction of a subset of p53 targets.^17^ Additionally, during myogenesis, Matrin3 is required for paraspeckle formation, likely by controlling adenosine to inosine (A-to-I) RNA editing of the *Ctn* RNA encoded by *SLC7A2*.^18^

Matrin3 knockout mice are embryonic lethal, indicating that it is required for normal development.^19^ Conditional deletion of Matrin3 in the neuronal lineage showed that it is essential for maintaining neuronal survival.^20^ Even though Matrin3 appears to be essential *in vivo*, disease-associated mutations were primarily found in neurodegenerative disorders, e.g., in familial amyotrophic lateral sclerosis (ALS) and frontotemporal dementia (FTD).^21^ The impaired formation of dynamic nuclear condensates due to ALS-associated mutations in Matrin3 possibly contributes to its pathogenic mechanism.^22^

There is conflicting evidence for a role of Matrin3 in cancer, indicating a potential role of Matrin3 as an oncogene or a tumor suppressor.^23–25^ While little is known about the role of Matrin3 in CRC, the cancer type our research focuses on, this nevertheless places Matrin3 among the many RBPs with potential oncogenic or tumor-suppressive roles. Typically, RBPs can bind multiple transcripts, and aberrant expression of a single RBP can affect the expression of a vast array of genes and abnormal phenotypes implicated in diseases including cancer.^26^ For instance, in CRC, the RBPs MSI1 and MSI2^27^ and SRSF1^28^ have oncogenic functions. In addition, the p53-induced RBP ZMAT3 has tumor-suppressive effects by functioning as a key splicing factor.^29,30^ It remains unclear which of the many Matrin3 RNA targets^11–13^ would mediate the function of Matrin3 in CRC or other types of cancer.

To understand the molecular mechanism(s) by which Matrin3 mediates its effects, we performed PAR-CLIP (photoactivatable ribonucleoside-enhanced crosslinking and immunoprecipitation), a UV crosslinking-based technique that identifies RBP target RNAs at nucleotide resolution on a transcriptome-wide scale.^31,32^ In HCT116 cells (CRC), Matrin3 binds to thousands of pre-mRNAs at pyrimidine-rich sequence elements, repressing inclusion of nearby exons. For functional analysis, we focused on one of the top misspliced targets upon Matrin3 knockdown, *CDC14B*, a key regulator of mitotic spindle assembly.^33^ We show that overexpression of CDC14B rescues the defects in mitotic spindles observed upon Matrin3 knockdown, suggesting that the effects of Matrin3 on mitotic spindle are mediated by CDC14B. Collectively, these data reveal a growth-promoting function of Matrin3 in CRC cells that is—at least partially—mediated through splicing changes of CDC14B.

## RESULTS

### Matrin3 binds to pyrimidine-rich intronic sites in nascent transcripts encoding proteins that regulate the mitotic spindle

**Figure 1.**
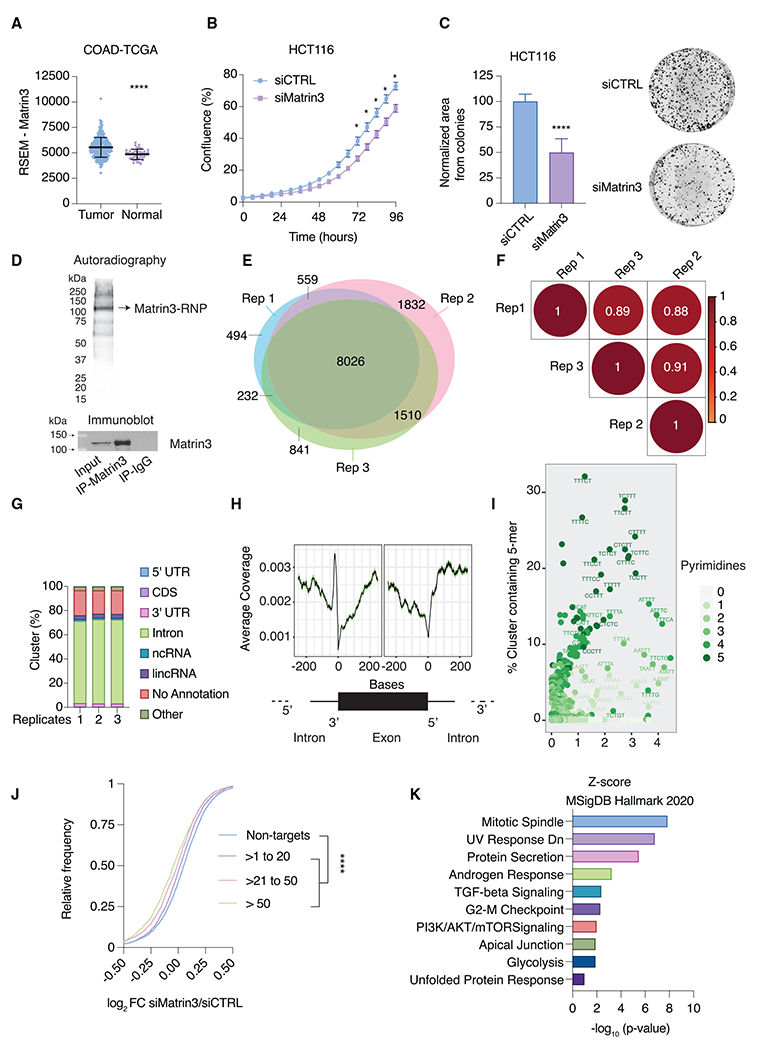
Matrin3 is a potential oncogene in colorectal cancer and binds pre-mRNAs to regulate their expression (A) Matrin3 mRNA expression in colorectal cancer (TCGA COAD) from the TSVdb database compared with normal colon tissues. Unpaired, two-sided t test; tumor, n = 287; normal, n = 41. (B) Cell proliferation assays after siRNA-mediated knockdown of Matrin3 in HCT116 cells. Two-way ANOVA test; n = 3; error bars: SEM, and *p < 0.05. (C) Colony formation in HCT116 cells after Matrin3 knockdown. Unpaired, two-sided t test; n = 3. (D) (Top panel) Representative figure of autoradiography of the P^32^-labeled Matrin3 RNP after immunoprecipitation (IP). (Bottom panel) Matrin3 immunoblot of input, Matrin3 IP, and immunoglobulin G (IgG) IP. (E) Intersection of Matrin3 target transcripts identified in three biological PAR-CLIP replicates. (F) Spearman’s correlation matrix of crosslinked sequence read frequency (measured by number of T-to-C mutations) on target transcripts for the PAR-CLIP replicates. (G) Average distribution of binding sites from Matrin3-bound RNAs across different annotation categories. (H) Metagene analysis of Matrin3 binding sites around the intron-exon boundary. (I) Scatterplot of *Z* scores (x axis) and frequency of occurrence (y axis) of all possible 5-mers in Matrin3 PAR-CLIP binding sites. Shades of green indicate the number of pyrimidines. (J) Cumulative distribution of gene abundance change (log_2_) determined by RNA-seq after Matrin3 knockdown and binned by normalized crosslinked reads per million (NXPM). Two-tailed Komogorov-Smirnov test; non-targets, n = 2,579; [1 to 20 NXPM] n = 2,701; [21-50 NXPM] n = 900; [>50 NXPM] = 1,015. (K) Plot of p values (−log_10_) of the enriched gene set enrichment analysis (GSEA) Molecular Signature Database (MSidDB) terms constructed from top Matrin3 targets. Error bars: SD, and ****p < 0.0001.

We first determined Matrin3 mRNA expression in a panel of human tumors compared with normal tissues. Matrin3 mRNA was significantly overexpressed in tumors, most frequently in CRC (Figures 1A, S1A, and S1B). Consistent with these data, Matrin3 knockdown resulted in decreased proliferation and clonogenicity in multiple cell lines (Figures 1B, 1C, and S1C–S1J), normal human diploid lung fibroblasts (IMR90 and WI38), and normal colon epithelial cells (HCEC-1CT) (Figures S1K–S1P). Interestingly, Matrin3 knockdown in HCT116 did not result in cell death or changes in the cell cycle (Figures S1S and S1T). Furthermore, in the DepMap database (Cancer Dependency Map: https://depmap.org/portal), comparing the effect of specific gene loss on growth across more than 1,000 cancer cell lines on proliferation, Matrin3 scored negative on average, compared with the expected negative and positive scores, respectively, for an essential control gene (PLK1) and a tumor-suppressor gene (p53) (Figures S1Q and S1R). These data suggest that Matrin3 promotes growth in diverse cell types.

Next, we mapped Matrin3 binding sites on RNAs on a transcriptome-wide scale at nucleotide resolution in HCT116 cells using 4-thiouridine (4SU) PAR-CLIP.^31,32^ For PAR-CLIP, we IPed endogenous, UV-crosslinked Matrin3 RNP. Autoradiography of crosslinked, ribonuclease-treated Matrin3 IP fractionated on denaturing protein gels revealed a prominent band migrating at the expected size of ~125 kDa, corresponding to the Matrin3 RNP (Figure 1D). We recovered the RNA fragments of Matrin3 RNPs and converted them into small RNA cDNA libraries for next-generation sequencing. Next, we determined clusters of overlapping reads that harbor characteristic T-to-C conversions diagnostic of 4SU crosslinking events at higher frequencies than expected by chance.^34^ For the three replicates, we found between 99,087 and 168,992 binding sites (Table S1). These binding sites were distributed on a set of >8,000 shared genes, suggesting that a significant proportion of expressed transcripts were bound by Matrin3 (Figure 1E). Overall, the biological replicates showed excellent correlation with a R^2^ of ~0.9 (Figure 1F). Approximately 70% of Matrin3 binding sites were found on intronic regions (Figure 1G), indicating that Matrin3 was interacting with nascent transcripts, consistent with its nuclear localization and previous CLIP-based studies.^11–13^ Some of these previous studies found a role for Matrin3 in alternative splicing, and indeed, in our data, Matrin3 bound broadly across nascent transcripts with a pronounced enrichment near the 3′ splice site (Figure 1H), most likely at the polypyrimidine tract required for splicing, considering that Matrin3 binding sites were enriched for 5-mer-containing pyrimidines (Figures 1I and S2A).

To investigate the gene regulatory roles of Matrin3, we next performed RNA sequencing (RNA-seq) from HCT116 transfected with Matrin3 small interfering RNAs (siRNAs) or a negative control siRNA. Knockdown of endogenous Matrin3 led to a modest but significant decrease in target mRNA levels. The magnitude of this effect was dependent on the overall strength of binding, i.e., the number of Matrin3 binding sites per target mRNA, or the number of crosslinked reads per target mRNA normalized by overall mRNA abundance (Figure 1J; Table S1). We previously found that both metrics correlated well with the occupancy of an RBP on its target.^35^

We next asked which basic architectural features^36^ differentiated Matrin3 targets from non-targets. Matrin3 targets were typically encoded on longer loci than non-targets. As one would expect from an RBP binding nascent transcripts, Matrin3 PAR-CLIP binding site numbers positively correlated with transcript length (Figure S2B), number of exons (Figure S2C), exon length (Figure S2D), or intron length (Figure S2E). Top Matrin3 targets had lower GC content compared with weaker targets or non-targets (Figure S2F), consistent with the preference of RRM domains binding unstructured RNA. Comparison of our Matrin3 PAR-CLIP (from HCT116) with Matrin3 enhanced CLIP (eCLIP) from HepG2 cells^37^ showed that they were reasonably similar, with a Spearman’s correlation coefficient of ~0.5 (Figures S2H and S2I), reflecting the different transcriptome of the cell lines, different CLIP-seq methodology, and our deeper dataset (Figure S2J). Pathway analysis showed that the top Matrin3 targets (>50 binding sites) were enriched for genes related to the mitotic spindle (Figure 1K), a structure important for mitosis. Thus, the PAR-CLIP interactome complemented our cell-based findings that Matrin3 knockdown resulted in reduced proliferation and clonogenicity.

### Matrin3 knockdown leads to inclusion of exons proximal to its binding sites

**Figure 2.**
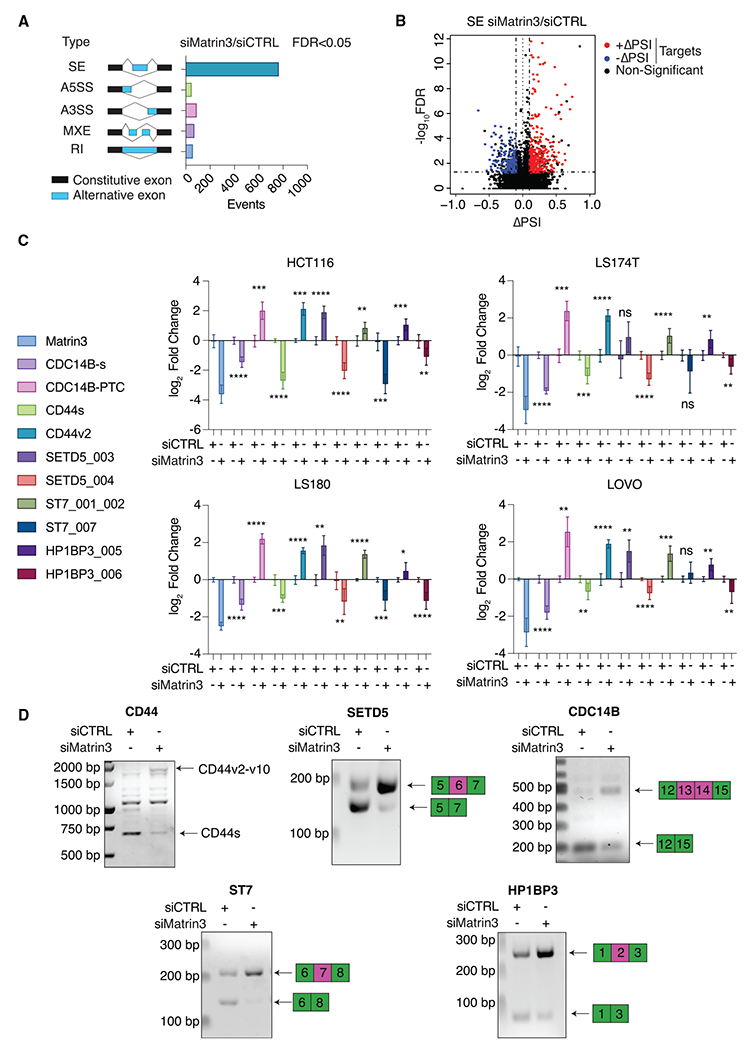
Matrin3 is a splicing repressor, and its knockdown results in widespread exon inclusion in target mRNAs (A) Bar plot categorizing the number of different alternative splicing events after Matrin3 knockdown in HCT116 cells (FDR < 0.05 and ΔPSI ≥ 10%). (B) Volcano plot of FDR (−log_10_) versus ΔPSI of exon skipping (SE) events upon Matrin3 silencing. Black dots, SE events with FDR >0.05; blue and red dots, exon exclusion and inclusion events, respectively, with FDR <0.05 in Matrin3 target mRNAs. (C) qRT-PCR for a panel of mRNAs; either full-length mRNA or inclusion levels of indicated exons were measured and normalized to GAPDH upon knockdown of Matrin3 in four different CRC cell lines (HCT116, LS174T, LS180, and LOVO). Unpaired, two-sided t test; n = 3. (D) Representative images (n = 3) of semi-qRT-PCR upon Matrin3 knockdown in HCT116 cells with primers designed around alternatively spliced exon(s) of genes showed in (C). Error bars: SD, and *p < 0.05, **p < 0.01, ***p < 0.001, ****p < 0.0001, and ns, non-significant.

Matrin3 has been previously characterized as a regulator of alternative splicing.^11–13^ Therefore, we analyzed alternative splicing (AS) patterns in our polyA-selected RNA-seq from HCT116 after Matrin3 knockdown. The majority of the AS events (false discovery rate [FDR] < 0.05, ΔPSI ≥ 10%) involved cassette exons or skipped exons (SEs) (Figures 2A and S2G). Of those events, >70% were found in Matrin3-bound RNAs. As reported previously in other cell types,^11,13^ Matrin3 acted mainly as a splicing repressor with ~66% of the SE events in Matrin3 targets resulting from exon inclusion after Matrin3 knockdown (Figure 2B).

We next validated some of these AS events on transcripts with high ΔPSI in HCT116 and three other CRC cell lines (LS174T, LS180, and LOVO) by qRT-PCR and in HCT116 cells also by semi-qRT-PCR. Specific exon inclusion events in the *CDC14B, CD44, SETD5, ST7,* and *HP1BP3* pre-mRNAs mirrored the RNA-seq results in all cases (Figures 2C, 2D, and S3). Taken together, our results indicate that Matrin3 binding close to 3′ splice sites suppresses AS and that its loss results in mRNA misprocessing that manifests in the inclusion of otherwise excluded exons.

### The growth-promoting function of Matrin3 is mediated, in part, by CDC14B

**Figure 3.**
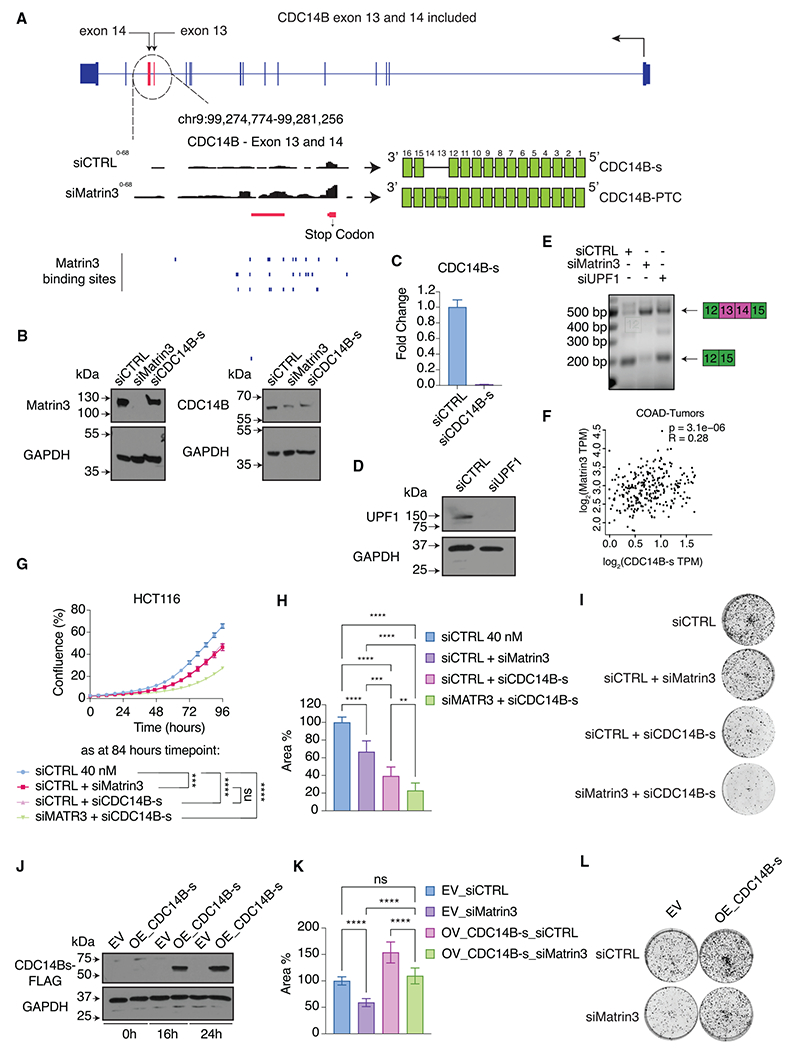
CDC14B alternative splicing is directly regulated by Matrin3 and partially mediates the growth-promoting effects of Matrin3 (A) Genome browser image of the CDC14B-s with exons 13 and 14 included. The focused region shows tracks of a sample treated with siCTRL or siMatrin3 in HCT116 cells. Lower tracks show Matrin3 RNA-binding sites in the region and the resultant RNA variants (CDC14B-s or CDC14B-PTC). (B) Immunoblots from HCT116 whole-cell lysates after siRNA-mediated knockdown of Matrin3 or CDC14B-s using antibodies against Matrin3 or CDC14B or GAPDH as loading control. (C) qRT-PCR quantifying CDC14B-s variant expression after knockdown of CDC14B-s using a control siRNA or a CDC14B-s siRNA. n = 2. (D) Immunoblots using an antibody against UPF1 or GAPDH as loading control from HCT116 whole-cell lysates after knockdown of UPF1. (E) Representative images (n = 3) of semi-qRT-PCR upon Matrin3 or UPF1 knockdown in HCT116 cells with primers designed in exons 12 and 15 of CDC14B. (F) Spearman’s correlation of Matrin3 and CDC14B-s expression in TCGA COAD tumor samples deposited in the Gepia2 database (http://gepia2.cancer-pku.cn/). (G) Incucyte live-cell proliferation assays after Matrin3 and/or CDC14B-s knockdown in HCT116 cells. Two-way ANOVA test; n = 3; error bars: SEM. (H) Colony-formation assays from HCT116 cells after Matrin3 and/or CDC14B-s knockdown. Unpaired, two-sided t test; n = 3; error bars: SD. (I) Representative images of colonies for the experiments in (H). (J) Immunoblotting was performed using an anti-FLAG or anti-GAPDH antibody after induction of CDC14B-s-FLAG expression with doxycycline at the indicated time points. EV refers to empty vector control and OE to overexpression of CDC14B-s. (K) Colony-formation assays from HCT116 cells overexpressing CDC14B-s-FLAG using a doxycycline-inducible system after Matrin3 knockdown. EV refers to empty vector control and OE to overexpression of CDC14B-s. Unpaired, two-sided t test; n = 3. (L) Representative images of colonies for the data in (K). Error bars: SD, and **p < 0.01, ***p < 0.001, ****p < 0.0001, and ns, non-significant.

Next, we chose to focus on *CDC14B* for functional analysis, as it was among the genes with most significantly changed ΔPSI upon Matrin3 knockdown. We hypothesized that the significant reduction in cell proliferation after Matrin3 knockdown is mediated, at least in part, by missplicing of *CDC14B. CDC14B* encodes a protein tyrosine phosphatase that is similar to *Saccharomyces cerevisiae Cdc14* and regulates interphase nuclear architecture, mitotic spindle assembly, and M phase exit.^33^ In all cell lines tested, Matrin3 knockdown resulted in ~60% reduction of the most abundant CDC14B variant in HCT116 cells (CDC14B-003 or ENST00000375241, only isoform detectable by long-read sequencing; Table S1), which we refer to as a “standard variant” or “CDC14B-s” (Figure 2C). The decrease in CDC14B-s levels after Matrin3 knockdown was accompanied by increased inclusion of two exons, which are only annotated in CDC14B-008 (ENST00000481149), a potential processed transcript variant that does not encode a protein. We refer to the longer transcript variant as the “PTC variant” or “CDC14B-PTC” (Figure 3A) because exon 13 introduces a premature termination codon (PTC), which makes this transcript a potential substrate for non-sense-mediated decay (NMD). Immunoblotting confirmed a decrease in CDC14B protein after Matrin3 knockdown (Figure 3B). We were also able to specifically knockdown the CDC14B protein and CDC14B-s transcript with an siRNA specifically targeting the CDC14B-s transcript (Figures 3B and 3C). Next, we validated that CDC14B-PTC is subject to NMD by knocking down UPF1, the core component of the NMD machinery, which indeed resulted in increased CDC14B-PTC abundance (Figures 3D and 3E).

In CRC, we observed a modest but significant positive correlation between Matrin3 and CDC14B-s mRNA levels (Figure 3F). Knockdown of Matrin3 or CDC14B-s resulted in a similar decrease of proliferation, and concurrent knockdown of Matrin3 and CDC14B-s had an additive effect on proliferation (Figure 3G). In colony-formation assays, knockdown of CDC14B-s alone had a stronger effect than knockdown of Matrin3, which was further augmented when both Matrin3 and CDC14B-s were depleted (Figures 3H and 3I), suggesting that additional Matrin3 targets affecting clonogenicity could mask the effect of CDC14B alone. Colony-formation assays from cells overexpressing CDC14B-s showed a modest but significant (****p < 0.0001) rescue in clonogenicity of Matrin3 knockdown cells (Figures 3J–3L). These data suggest that CDC14B splicing regulation contributes to the decreased growth observed upon Matrin3 knockdown.

### Matrin3 regulates microtubule dynamics, mitotic spindle orientation and bipolarity via CDC14B

**Figure 4.**
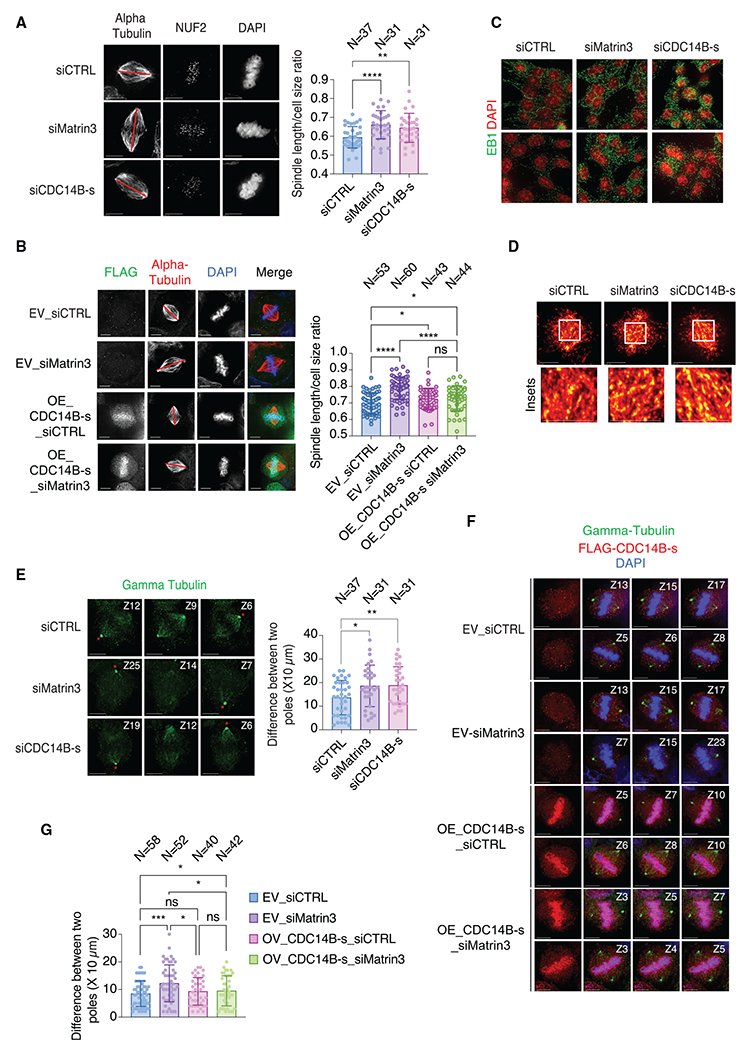
CDC14B overexpression rescues the effects of Matrin3 knockdown on microtubule dynamics and spindle orientation (A) Representative immunofluorescence images of MG132-treated HCT116 cells in metaphase immunostained for α-tubulin (spindle microtubules) or Nuf2 (kinetochores) after transfection with siRNAs against Matrin3, CDC14B-s, or control. Nuclei were stained with DAPI, Scale bar, 5 μm; red bars indicate length of mitotic spindles. (Right panel) Bar graph of ratio between spindle length and cell size was calculated. “N” denotes number of cells analyzed. Unpaired, two-sided t test. (B) Representative immunofluorescence images of MG132-treated HCT116 cells in metaphase immunostained for CDC1B-s-FLAG using anti-FLAG or α-tubulin (spindle microtubules) (left panel) after induction of CDC1B-s-FLAG with doxycycline and transfection with Matrin3 siRNAs or control siRNA. Nuclei were stained with DAPI, Scale bar, 5 μm; red bars indicate length of mitotic spindles. (Right panel) Bargraph of ratio between spindle length and cell size was calculated. “N”, number of cells. Unpaired, two-sided t test. (C) Representative immunofluorescence images showing EB1 comet density in interphase of HCT116 cells transfected with siRNAs as indicated and immunostained for EB1 and stained with DAPI for nuclei. Scale bar, 5 μm. n = 2. (D) Representative immunofluorescence images of MG132-treated cells in metaphase showing EB1 comets in cells treated with siRNAs as indicated and immunostained for EB1 and images pseudocolored to show highly intensified comets as yellow and less intensified comets as red. Scale bars, main image, 5 μm; insets, 1 μm. n = 2. (E) Representative immunofluorescence images of MG132-treated cells in metaphase showing mitotic spindle orientation as depicted by planes of two spindle poles denoted with red asterisks in HCT116 cells transfected with the indicated siRNAs and stained for γ-tubulin (left panel). Cells were imaged with multiple 10 μm z stacks, and the numbers of z stacks between planes of two poles were measured in each siRNA condition and plotted as a bar plot (right panel). “N” denotes number of cells analyzed from two independent experiments. Unpaired, two-sided t test. (F) Representative immunofluorescence images of MG132-treated HCT116 cells in metaphase showing mitotic spindle orientation as depicted by planes of two spindle poles in HCT116 cells transfected with the indicated siRNAs and immunostained for CDC1B-s-FLAG using anti-FLAG or γ-tubulin (spindle poles). Cells were imaged with multiple 10 μm z stacks, and the numbers of z-stacks between planes of two poles were measured in each siRNA condition and plotted as a bar plot (right panel). “N”, number of cells from two independent experiments. (G) Quantification of the difference between two poles depicted in (F). Unpaired, two-sided t test. Error bars: SD, and *p < 0.05, **p < 0.01, ***p < 0.001, ****p < 0.0001, and ns, non-significant.

CDC14 is a conserved protein in eukaryotes and its crucial role in mitosis in budding yeast^38^ prompted us to examine if Matrin3 affects mitotic spindle architecture via regulation of *CDC14B.* To visualize the architecture of the mitotic spindle, cells were immunostained with alpha-tubulin for spindle microtubules and Nuf2 as an outer kinetochore marker. We found that in metaphase, mitotic spindles were slender and significantly elongated upon knockdown of Matrin3 or CDC14B-s (Figure 4A). CDC14B-s overexpression significantly rescued the elongated and misoriented mitotic spindles observed in Matrin3 knockdown cells (Figure 4B), suggesting that the observed defects in spindle length upon Matrin3 knockdown are largely mediated by defects in CDC14B splicing.

We reasoned that the elongated spindles could be caused by altered microtubule dynamics with more polymerizing microtubules present, as seen in cells with more stabilized microtubules.^39^ Therefore, we examined the density of EB1 comets that mark the plus ends of polymerizing microtubules in interphase. Qualitative analysis showed that in interphase cells, the density of EB1 comets was increased upon Matrin3 or CDC14B-s knockdown (Figure 4C), suggesting that the density of polymerizing microtubules is higher. Additionally, EB1 intensity measured by imaging and the pseudocoloring EB1 signal showed brighter comets in Matrin3 or CDC14B-s knockdown cells (Figure 4D), confirming the presence of highly polymerized microtubules.

During symmetric cell division in epithelial cells, microtubule dynamics help orient mitotic spindles parallel to the plane of attachment.^40^ Matrin3- or CDC14B-s-depleted cells showed misorientation of mitotic spindles, with higher z stack counts and presence of two poles of a mitotic spindle on different optical planes (Figure 4E). 3D-reconstructed images clearly visualized this misorientation of mitotic spindles (Videos S1, S2, and S3). CDC14B-s overexpression significantly rescued the misoriented mitotic spindles observed in Matrin3 knockdown cells (Figures 4F and 4G), suggesting that the observed defects in mitotic spindle orientation upon Matrin3 knockdown are largely mediated by defects in CDC14B splicing.

A previous study showed that after depletion of the microtubule depolymerizer MCAK, cells fail to form bipolar spindles.^41^ Therefore, we transiently changed the mitotic spindle geometry by treating cells with monastrol, an Eg5 inhibitor that inhibits mitotic spindle bipolarity,^42^ followed by monastrol washout and arresting cells in MG132 to capture cells in bipolar metaphase.^43^ We found that proportions of monopolar mitotic cells were significantly increased in Matrin3 or CDC14B-s knockdown cells (Figures S4A and S4B), suggesting a severe delay in mitotic spindle bipolarity. A delay in spindle bipolarity could lead to defects in chromosome congression,^43^ and indeed, the proportion of cells with uncongressed chromosomes was increased in asynchronous Matrin3 or CDC14B-s knockdown cells (Figure S4C). Chromosome congression defects often lead to chromosome segregation defects, especially if the chromosome uncongression was due to defects in kinetochore-microtubule attachments^44^ or inefficient spindle assembly checkpoint.^45^ Nevertheless, we did not observe significantly increased chromosome segregation defects in Matrin3 or CDC14B knockdown cells (Figure S4D), suggesting that in Matrin3 or CDC14B-s knockdown cells, chromosomes eventually congress in metaphase and segregate normally, albeit with a delay. We propose that the delay in mitotic spindle bipolarity and chromosome congression could contribute to the decreased proliferation observed upon Matrin3 or CDC14B knockdown.

We arrive at the mechanistic model that Matrin3 contributes to maintenance of microtubule dynamics, spindle morphology, and proper mitotic spindle orientation by regulating CDC14B-s abundance and suggest that defects in these processes may contribute to the reduced proliferation of cells after knockdown of Matrin3.

## DISCUSSION

Matrin3 is typically studied in the context of neurodegenerative disorders, like ALS.^46^ Here, we discovered that Matrin3 promotes CRC cell growth by suppressing CDC14B splicing, resulting in altered microtubule dynamics.

Although several Matrin3 CLIP experiments were reported previously,^11–13^ most of these reports focused on the relationship of the binding sites of Matrin3 and PTBP1, another splicing regulator. By integrating RNA-seq and PAR-CLIP data, we corroborated that Matrin3 acts mainly as a splicing repressor.^11,13^ We do not necessarily interpret this as evidence for Matrin3 being a regulatory molecule but rather as it being required for proper mRNA processing. Consistently, we found that Matrin3 coats its top targets across the entire pre-mRNA (exons and introns) and that loss of Matrin3 results in reduced target mRNA abundance, likely by cytoplasmic degradation pathways dedicated to sensing misprocessed and PTC-bearing mRNA, like NMD.^47^ Nevertheless, many of the exon inclusion events upon Matrin3 knockdown will still result in alternatively spliced transcripts.

Matrin3 top targets were enriched for genes related to mitotic spindle formation and regulation and included CDC14B, which is important for mitosis.^48,49^ Our data demonstrate that Matrin3-dependent down-regulation of the CDC14B-s variant affected proliferation and phenocopied Matrin3 knockdown. Because we found that CDC14B-s overexpression could rescue the effect of Matrin3 knockdown on clonogenicity, mitotic spindle length, and orientation, we concluded that CDC14B is one of the critical targets responsible for mediating these phenotypes.

We observed that Matrin3 represses the inclusion of exons 13 and 14 of the CDC14B mRNA, likely by coating splice sites on the nascent mRNA and preventing co-transcriptional splicing inclusion^50,51^ of these exons and promoting the formation of the canonical CDC14B-s isoform. When included, exon 13 includes a PTC, marking the longer CDC14B-PTC transcript for degradation via NMD. We focused on CDC14B-s because the CDC14B-PTC is of low abundance in our RNA-seq data, consistent with it being a potential NMD target. Nevertheless, a noncoding, shorter transcript including exons 13 and 14 is found in databases (ENST00000481149), even though we do not find evidence of its existence by analysis of long-read sequencing (Isoseq) data from HCT116 cells.

Other studies also observed that Matrin3 loss results in reduced cell proliferation^24,25,52^ and suggested increased apoptosis as a cause. Our data indicate that at least in CRC cells, Matrin3 knockdown results in increased polymerizing spindle microtubules, delayed mitotic spindle bipolarity, and delayed chromosome congression, possibly leading to reduced cell proliferation, as studies have shown that the stabilization of microtubules inhibit cell proliferation.^53–55^ Functional studies of human CDC14B protein had conflicting results regarding its role in mitosis. For instance, Berdougo et al.^33^ generated cell clones with non-functional CDC14B protein and showed that it did not alter the proliferation or cause mitotic spindle defects. On the other hand, Tumurbaatar et al.^49^ found that CDC14B knockdown reduced cell proliferation, increased the amount of bi- and multinucleated cells, and resulted in impaired metaphase-anaphase transition and caused mitotic delay that resulted in cell death. We observed an increased proportion of cells with monopolar spindles following monastrol washout and uncongressed chromosomes upon knockdown of Matrin3 or CDC14B-s. We rule out that these phenotypes are due to defects per se in mitotic spindle bipolarity or chromosome congression, as we observe a mitotic delay rather than chromosome segregation defects. Other reports demonstrated that CDC14B can have an oncogenic effect through distinct mechanisms. Examples of these mechanisms include the activation of the Ras-Raf-Mek pathway, which mediates the oncogenic effect of CDC14B,^56^ and also the degradation of p53, which is promoted by CDC14B phosphatase activity.^57,58^

Matrin3 may be one of several RBPs regulating microtubule dynamics. Previously, the RNA-binding properties of the classical end-binding protein EB1,^59^ as well as of the adenomatous polyposis coli (APC)^60^ scaffolding proteins, were found to be required for their function, suggesting an intimate relationship between RNA regulation and microtubule dynamics. In conclusion, we found that Matrin3 regulates splicing of CDC14B, leading to increased expression of CDC14B-s, which promotes destabilization, shorter length, and proper orientation of microtubules, that culminates in more events of mitosis and, consequently, CRC cell proliferation.

### Limitations of the study

We found that the Matrin3 regulates microtubule dynamics, mitotic spindle orientation, and bipolarity by repressing CDC14B pre-mRNA splicing. Although we validated the regulation of CDC14B pre-mRNA splicing by Matrin3 and decreased growth upon Matrin3 knockdown in multiple cell lines, the effects on the microtubule dynamics and the mitotic spindle upon Matrin3 knockdown were not validated in multiple cell types. We also found that the decreased clonogenicity observed upon Matrin3 knockdown was only partially rescued upon CDC14B overexpression in Matrin3 knockdown cells. This suggests that regulation of CDC14B splicing by Matrin3 is not the only mechanism by which Matrin3 functions to promote growth in CRC cells. In addition to CDC14B, several Matrin3 targets involved in regulation of cell cycle were identified in our PAR-CLIP and RNA-seq data. These targets remain to be validated at the pre-mRNA and protein levels and to be mechanistically dissected. Given that Matrin3 is essential for normal development in mice,^19^ future studies could determine the key functional targets of Matrin3 in mouse cells and tissues. Finally, it would be important to determine if specific Matrin3-bound RNAs identified in our study also play a role in diseases other than cancer such as neurodegenerative disorders, where Matrin3 mutations have been primarily found.^21^

## STAR★METHODS

### RESOURCE AVAILABILITY

#### Lead contact

Further information and requests for resources and reagents should be directed to and will be fulfilled by the lead contact, Ashish Lal (ashish.lal@nih.gov).

#### Materials availability

Cell lines and plasmids generated in this study are available from the lead contact upon request. All unique/stable reagents generated in this study are available from the lead contact with a completed materials transfer agreement.

#### Data and code availability

- The RNA-seq, Iso-Seq and PAR-CLIP data are available at Gene Expression Omnibus (GEO) and are publicly available as of the date of publication. Accession numbers are listed in the key resources table.
- This paper does not report original code.
- Any additional information required to reanalyze the data reported in this paper is available from the lead contact upon request.

### EXPERIMENTAL MODEL AND SUBJECT DETAILS

HCT116, LS180, LS174T and LOVO colorectal carcinoma cell lines; U2OS sarcoma cell line; WI38 and IMR90 normal fibroblast cell lines were purchased at American Type Culture Collection (ATCC). The HCEC-1CT normal colon cells were purchased from Evercyte. All cell lines were cultivated in DMEM medium (Gibco, Catalog no. 11965118) containing 10% Fetal Bovine Serum (FBS) (Gibco, Catalog no. 10082147) and 100 U/ml of penicillin and 0.1 mg/ml of streptomycin (Gibco, Catalog no. 15140122). Cells were grown at 37°C and 5% CO_2_. Cell lines were regularly checked for mycoplasma contamination using Venor^™^ GeM Mycoplasma Detection Kit (Sigma-Aldrich -Aldrich Co., Catalog no. MP0025-1KT).

### METHOD DETAILS

#### siRNA transfection

Cells were reverse transfected using siRNAs (final concentration 20 nM) and Lipofectamine^™^ RNAiMAX Transfection Reagent (Invitrogen^™^, Catalog no. 13778075) with slight modifications from the manufacturer’s protocol. When transfections were done to purify RNA for qPCR, 1.3 to 1.75 × 10^5^ cells/per well were seeded in 12-well plates adding a mixture that was pre-incubated at room temperature for 20 minutes and contained 2 μl of lipofectamine and 1 μl of 20 μM of siRNA in 200 μl of Opti-MEM^™^ (Gibco, Catalog no. 31985062). The mixture was added to 800 μl of complete medium and cells.

Cells prepared for RNA-seq, immunoblotting or colony formation assays were reverse transfected in 6 well plates. Briefly, 2 x 10^5^ to 3.5 x 10^5^ cells/per well were seeded with a solution containing 5 μl lipofectamine and 2.5 μl of a 20 μM siRNA in 500 μl of Opti-MEM^™^. The mixture was added to 2 ml of complete medium and cells.

Cells prepared for immunoblotting in Figures 3B and 3D were transfected twice. The second round of transfection was conducted 72 hours after the first transfection and harvested 72 hours after the second transfection.

Allstars Negative Control siRNA (Qiagen, Catalog no. 1027281) were used as control siRNAs. We used SMARTPool siRNAs (Horizon Discovery, Catalog no. L-017382-00-0005) against Matrin3 or (Horizon Discovery, Catalog no. L-011763-00-0005) UPF1. In order to target CDC14B standard variant, we designed an siRNA specific for the junction of the exons 12 and 15 of it (according to the model in the Figure 3A). The abovementioned siRNA, siCDC14B-s, was purchased from Integrated DNA Technologies (IDT). For proliferation and colony formation assays using those siRNAs, cells were transfected using siRNAs against more than one target (e.g., siCTRL and siMatrin3). Consequently, the final concentration of siRNAs was 40 nM Table S3 contains the sequences of custom siRNAs used.

#### Plasmid construction and generation of stable cell lines

cDNA sequence for the ENST00000375241 isoform of CDC14B-s was downloaded from the UCSC Genome Browser (GRCh38/hg38). The corresponding DNA sequence with linker and 3X-FLAG were synthesized using Twist Biosciences. The DNA fragments were PCR amplified and assembled into doxycycline inducible lentiviral vector using Gibson assembly.^73^ The doxycycline inducible vectors with blasticidin selection marker (pCW-Cas9-Blast_empty vector and pCW-Cas9-Blast_CDC14B-FLAG) were obtained from Addgene (Addgene Catalog. no. 83481) and subsequently digested with NheI/BamHI for cloning purposes. The final constructs were verified using Sanger sequencing.

The plasmids were expanded in DH5α cells (Invitrogen, Catalog no. 18265017) and purified with Monarch plasmid miniprep kit (NEB, Catalog no. T1010L). They were cotransfected into 293T cells together with a third generation of lentivirus packaging system using Lipofectamine 2000 (Thermo Fisher, Catalog no. 11668027). HCT116 cells were transduced and after 2 days the cells were selected with 10 μg/mL of blasticidin (Thermo Fisher, Catalog no. A1113903) for 1 week at a MOI of ~0.5.

#### RNA extraction, RT-qPCR and RT-PCR

Cells used for expression analysis by RT-qPCR were washed after 48 hours of transfection or after 72 + 72 hours for two rounds of transfection using DPBS 1X (Gibco, Catalog no. 14190250) and lysed using 250 or 500 μl (for 12 and 6-well plates, respectively) of TRIzol^™^ Reagent (Invitrogen^™^, Catalog no. 15596018) 48 hours post transfection. RNA was extracted according to the manufacturer’s protocol.

500 or 1000 ng of RNA was reverse transcribed using iScript^™^ Reverse Transcription Supermix (Biorad, Catalog no. 1708841). To the resulting 10 μl of cDNA, were added 45 or 90 μl of nuclease-free H_2_O, for the 500 or 1000 ng of RNA used in the reaction, respectively. 2.5 μl of diluted cDNA was used in the qPCR reaction together with 5 μl of FastStart Universal SYBR Green Master (Rox) (Millipore Sigma, Cat no. 4913914001), 0.2 μM (final concentration) of each primer and nuclease-free H_2_O enough for a 10 μl reaction and run on a StepOnePlus^™^ Real-Time PCR (Applied Biosystems^™^). We used GAPDH to normalize the expression and the relative expression was calculated using the 2^−ΔΔCt^ method^74^.

Semi-quantitative RT-PCRs were performed using 1μl of non-diluted cDNAs, 12.5 μl of Phusion^®^ High-Fidelity PCR Master Mix with HF Buffer (NEB, Catalog no. M0531S) 0.8 μM of each primer (final concentration) and nuclease-free H_2_O enough for a 25 μl of reaction. The PCR cycle used was as follows: 94°C for 5 minutes for 1 X, 98°C for 10 seconds, 50 to 60°C (CDC14B: 50°C; ST7: 56°C; CD44 and SETD5: 57°C; HP1BP3: 60°C) for 30 seconds and 72°C for 0.5 or 2 minutes (2 minutes for CD44, 1 minute for CDC14B and 0.5 minute for the rest of primers) for X28. The PCR products were run on 1 to 2% agarose gels.

Table S3 contains the sequences of primers used.

#### Immunostaining

For immunostaining, HCT116 parental or with inducible overexpression of CDC14B-S-FLAG or empty vector cells were transfected with siRNAs as described above, using 1.5 x 10^5^ cells/per well that were seeded on coverslips (Corning, Catalog no. 2850-18) inside 6-well plates. To the cells with inducible overexpression of CDC14B-s-FLAG or empty vector was added doxycycline at final concentration of 1 μg/ml the day before the transfection and at the day of transfection. After 48 hours of transfection, cells were fixed with ice-cold methanol for 1 minute. In case we wanted to observe cells in metaphase, we treated the cells with MG132 (Sigma Aldrich, Catalog no. 474790-10MG) at 10 μM for 90 minutes prior to fixing them.

Fixation was followed by blocking with 1% BSA in PBS/0.1% Tween (PBST) for 45 minutes at room temperature. Cells were incubated in primary antibodies for 3 hours at room temperature, washed 3 times in PBST and incubated with secondary antibodies and DAPI for 1 hour at room temperature. Following 3 washes with PBST, cells were mounted on slides using ProLong^™^ Gold Antifade Mounting media (Thermo Fischer, Catalog no. P36935). Mouse anti-CENP-A (Abcam, Catalog no. ab13939), rabbit anti-NUF2 (Abcam, Catalog no. ab176556), rabbit anti-Matrin3 (Bethyl Laboratories^®^, Catalog no. A300-591A) and mouse anti-EB1 (BD Biosciences, Catalog no. 610534) were used at 1:500 dilutions. Mouse anti-alpha-tubulin (Abcam, Catalog no. ab7291) and rabbit anti-gamma-tubulin (Abcam, Catalog no. ab11317) were used at 1:1000 dilutions. The secondary antibodies, goat anti-rabbit DY 488 (Thermo Fischer, Catalog no. 35552), goat anti-rabbit DY 594 (Thermo Fischer, Catalog no. 35560), goat anti-mouse DY 488 (Thermo Fischer, Catalog no. 35502), goat anti-mouse DY 594 (Thermo Fischer, Catalog no. 35510) were used at 1:500 dilution. DAPI was used at 1:5000 dilution.

#### 3D-reconstructed images

3D reconstructed images derived from cells treated with MG132 immunostained cells with anti-CENP-A, anti-gamma-tubulin and DAPI showed in figure 5D were imaged with multiple 10 μm z-stacks and 3D rendering was performed with Imaris version 9.5.0.

#### Immunoblotting

Cells used for immunoblotting were lysed using 200 μL of RIPA buffer (Life Technologies, Cat no. 89901) and sonicated for 5 seconds three times at power set of 50% (VirTis VIRSONIC 100). The lysates were centrifuged for 10 minutes at 4°C at 16,000 x g, and the supernatant was collected. Total protein was quantified using Pierce^™^ BCA Protein Assay Kit (Thermo Fischer, Catalog no. 23225) according to the manufacturer’s protocol. 10 to 20 μg of the lysate was loaded into (6% or 10%) SDS-polyacrylamide gels or precast gels Novex^™^ WedgeWell^™^ 4 to 20% (Thermo Fischer, Catalog no. XP04205BOX).

Proteins were transferred to a PVDF membrane using a semi-dry transfer apparatus (BioRad). The membrane was blocked for 1 hour using TBST (Tris-Buffered Saline - 19.98 mM Tris, 136 mM NaCl and Tween 0.05%, pH 7.4) containing 5% milk. We used the following primary antibodies: rabbit anti-Matrin3 (Bethyl Laboratories^®^, Catalog no. A300-591A) at 1:2000 dilution; rabbit anti-GAPDH (Cell Signaling, Catalog no. 5174S) at 1:3000 dilution; rabbit anti-CDC14B (Thermo Fischer, Catalog no. 34-8900) at 1:500 dilution and goat anti-UPF1 (Bethyl Laboratories^®^, Catalog no. A300-038A) at 1:2000 dilution. The primary antibodies were incubated overnight, except for GAPDH (1 hour of incubation at room temperature). The membranes were developed after 1 hour of secondary antibody incubation at 1:5000 dilution by using ECL^™^ Prime Western Blotting Detection Reagent (Fisher Scientific, Catalog no. RPN2232).

#### Monastrol wash out assays

Following siRNA transfections for 48 hours, cells were treated with 10 μM monastrol for 3 hours, followed by washing off monastrol and growing them in monastrol free fresh media containg 10 μM MG132 for 45 minutes to capture cells in bipolar metaphase. Cells were then fixed and immunstained with antibodies against alpha-tubulin to visualize mitotic spindles and CENP-A to visualize centromeres. Cells were imaged to quantify cells with monopolar (failed to form bipolar) and bipolar spindles.

#### Immunoprecipitation

Cells prepared for immunoprecipitation (IP) were lysed as described above. 25 μl of Pierce^™^ Protein A/G Magnetic Beads (Thermo Fischer, Catalog no. 88803) were prepared by washing them twice with PBS 1X and RIPA buffer once. 2 μg of anti-Matrin3 or normal rabbit IgG (Cell Signaling, Catalog no. 2729S) were coupled with the beads overnight. The next day the beads were washed again with RIPA buffer and 500 μg of lysate was used for IP for 4 hours. After that, the beads were washed five times with RIPA buffer, added to SDS-PAGE sample buffer and boiled at 95°C for 5 minutes. The same was done for the input sample. Samples were loaded into a 7.5% SDS-polyacrylamide gels for Immunoblotting using anti-Matrin3 antibody.

#### Colony formation assays

For this assay, cells were transfected with siRNAs twice. The second transfection was done 48 hours after the first one. Cells were seeded after 24 hours of the second transfection. 2 x10^3^ to 3 x 10^3^ cells/per well were seeded in 6-well plates for HCT116, LS180, U2OS and HCEC-1CT. In the case of HCT116 cells overexpressing CDC14B-s-FLAG or empty vector in an inducible system, doxycycline enough for 1 μg/ml was added 24 hours after seeding them. After 7 to 8 days, cells were fixed with ice cold methanol for 15 minutes and stained with crystal violet 0.5% in methanol (10%) for 15 minutes. The ImageJ (version 2.0.0-rc-43/1.52n) software package was used to analyze images of the area of colonies.

#### Proliferation assays

For proliferation assays, 5 x 10^2^ to 3 x 10^3^ cells/per well were seeded for HCT116, LS180, LS174T, IMR90 and WI38, in 96 well plates after 24 hours of transfection. Cells were incubated on Incucyte^®^ S3 Live-Cell Analysis Instrument and photographed each 6 hours for at least 4 days. The pictures were analyzed by measuring the occupied area (% confluence) of cell images over time with the software from the manufacturer’s device.

#### RNA-seq

RNA-Seq by poly (A) capture was performed in biological triplicates from HCT116 cells after 2 rounds of transfection of siMatrin3 or siCTRL siRNAs. The second transfection was done 72 hours after the first transfection. After 48 hours from the second transfection, cells were reseeded, and RNA was purified after 3 days from the second transfection using the RNeasy Plus Mini Kit (Qiagen, Catalog no. 74134) following the manufacturer’s instructions. Samples were sequenced on HiSeq4000 using Illumina TruSeq Stranded mRNA Library Prep and paired-end sequencing.

Total RNA-seq by ribosomal RNA knockdown was also performed in biological triplicates from HCT116 cells after 48 hours of siMatrin3 or siCTRL siRNAs transfection. Total RNA was extracted using TRIzol^™^ Reagent (Invitrogen, Catalog No. 15596018) according to manufacturer’s instructions. We used the NEBNext Ultra Directional RNA Library Prep Kit for Illumina (NEB, Catalog No. E7760) with NEBNext rRNA Knockdown Kit (NEB, Catalog No. E6318). The samples were sequenced on an Illumina HiSeq 3000 machine using the 50 cycles single end sequencing protocol.

Sequence reads were aligned with the reference genome (Human - hg19) and the annotated transcripts using STAR (version 2.5.4a)^64^ or TopHat (version 2.1.1)^63^ for poly (A) capture or ribosomal knockdown sequencing, respectively. The gene expression quantification analysis was performed for all samples using RSEM (version 1.2.31)^65^. Differential gene expression was quantified using DESeq2 (version 1.26.0).^66^

For transcript analysis, fastq files from poly (A) sequencing were trimmed using Trimmomatic (version 0.36)^67^ and Trim Galore (version 0.4.5) (https://www.bioinformatics.babraham.ac.uk/projects/trim_galore/). Gencode v19 version transcripts from were quantified using Salmon (version 0.14.1).^68^ The normalized TPM expression values were obtained from the Sleuth package (version 0.30.0).^69^

#### Isoform-sequencing (Iso-Seq)

RNA from HCT116 cells was purified using the RNeasy Plus Mini Kit (Qiagen, Catalog number 74134) following the manufacturer’s instructions. The library was prepared using Iso-Seq^™^ Express Template Preparation protocol (Pacific Biosciences, CA, USA) for transcripts <2 kb. Raw subreads generated with PacBio SMRTlink (smrtlink-release_9.0.0.92188) were converted into HiFi circular consensus sequences (CCS). Demultiplexing was done using PacBio IsoSeq v3. The FLNC reads (Full-Length Non-Concatemer) were mapped to hg38 using Minimap2 software.^71^ Isoforms classification was done using SQANTI3.^72^

#### PAR-CLIP

Matrin-3 PAR-CLIP method was performed in three biological replicates as described previously.^31,61^ Below is a summary of the protocol used:

##### A. Cell culture and UV crosslinking

HCT116 cells were seeded in 15 mm dishes and were treated with 100 μM final concentration of 4-Thiouridine (Sigma-Aldrich, Catalog no. T4509) when they reached ~80% of confluency. For each sample, the cells were grown in 10 dishes. After 16 hours of treatment, the medium was removed, and cells were crosslinked for 5 min with 312 nm UV light using a Spectrolinker XL-1500 (Spectronics Corporation). Cells were scraped on ice using a rubber policeman and cold DPBS 1X, after which they were centrifuged at 500 xg for 5 min at 4°C. The cells were washed with DPBS 1X, centrifuged as above mentioned and the pellet was frozen at −80°C until further use.

The cell pellets were thawed on ice and cells were lysed using 1.5 volumes of RIPA buffer (150 mM NaCl, 1.0% NP-40, 0.5% sodium deoxycholate, 0.1% SDS (sodium dodecyl sulfate) and 50 mM Tris, pH 8.0 and 1 tablet of complete^™^, EDTA-free Protease Inhibitor Cocktail (Millipore Sigma, Catalog no. 11836170001) per 10 ml of solution). Subsequently, the cells were sonicated (VirTis VIRSONIC 100) 3 times for 30 seconds each (power set to 60%) and left on ice for 15 min. The lysates were cleared by centrifugation at 16,000 x g for 15 min at 4°C.

##### B. Immunoprecipitation, RNase T1 treatment and dephosphorylation

The lysates were treated with RNase T1 (1 U/μl) (Thermo Fisher, Catalog no. EN0541) for 15 min at room temperature, and then cooled on ice for 5 min 80 μL of Pierce Protein A/G Magnetic Beads (Thermo Fisher, Catalog no. 88803) coupled with 20 μg of anti-Matrin3 antibody (Bethyl Laboratories, Catalog No. A300-591A) were added to the lysates and they were incubated at 4°C for 4 hours with rotation.

The beads were washed 3 times with 1 mL IP buffer (20 mM Tris, pH 7.5 150 mM NaCl, 2 mM EDTA, 1% (v/v) NP40 and 0.5 mM DTT (added fresh)), resuspended in 80 μL of IP buffer with RNase T1 (10 U/μl) for 15 min at room temperature with rotation. The beads were washed 3 times with 1 ml IP buffer, resuspended in 80 μl of dephosphorylation buffer (50 mM Tris-HCl, pH 7.9, 100 mM NaCl 10 mM MgCl_2_ and 1 mM DTT) with Alkaline Phosphatase, Calf intestinal (CIP) (NEB, Catalog no. M0290S) (0.5 U/μl) for 10 min at 37°C with shaking. Beads were washed 2 times with 1 ml of dephosphorylation buffer, then 2 times with 1 ml of PNK buffer without DTT (50 Mm Tris-HCl, pH 7.5, 50 mM NaCI and 10 mM MgCl_2_). Next, the beads were added to 80 μl of PNK buffer containing DTT (70 mM Tris-HCl, pH 7.6, 10 mM MgCl_2_ and 5 mM DTT).

##### C. Phosphorylation with γ-^32^P-ATP of crosslinked RNA, SDS-PAGE and Proteinase K digestion

The resuspended beads were added to γ-^32^P-ATP (0.5 μCi) and T4 PNK kinase (NEB, Catalog no. M0201S) (1 U/μl) and the beads were incubated at 37°C for 30 min with shaking. Next, non-radioactive ATP (100 μM) was added to the reaction and the beads were incubated for more 5 min at 37°C with shaking. Next, the beads were washed 5 times with 1 ml of PNK buffer without DTT, resuspended in 70 μl of 2x SDS-PAGE loading buffer, boiled for 5 min at 95°C, and vortexed.

The recovered material was loaded onto an SDS-Polyacrylamide gel NuPAGE^™^ 4 to 12%, Bis-Tris, 1.0 mm, Midi Protein Gel, 12+2-well (Thermo Fisher, Catalog no. WG1401BOX) and after the run the gel was transferred to a nitrocellulose membrane using a semi-dry transfer apparatus. Next, the membrane was wrapped with plastic film and exposed to a blank phosphorimager screen overnight at −20°C.

The next day, the screen was scanned in a phosphorimager imager and the band correspondent to 125 kDa was cut. The membrane pieces were added to 1.5 ml tubes and ribonucleoproteins were digested for 30 minutes initially in 200 ml of Proteinase K buffer (50 mM Tris-HCl pH 7.5, 75 mM NaCl, 6.25 mM EDTA and 1% (w/v) SDS) containing 1.2 mg/ml of Proteinase K (Roche, Catalog no. 03450376103) and then 150 μl of the same buffer including 0.75 mg/ml of Proteinase K was added twice at each 30 minutes, completing 1 hour and 30 minutes of reaction. The recovered RNA was purified with phenol-chloroform extraction and ethanol precipitation. The RNA pellet was resuspended in 9 μl of water.

##### D. 3’ adapter ligation

For the ligation of 3’ adapter to the recovered RNA, a mix was made with 2 μl RNA ligase buffer without ATP (500 mM Tris-HCl, pH 7.6, 100 mM MgCl_2_ and 10 mM DTT), 6 μl 50% PEG-8000, 2 μl of 50% adenylated 3′ adapter and added to the RNA. The resultant solution was boiled for 1 min at 90°C. The same was done for the 19/35 nt RNA marker mix (see item 2.2 in in Benhalevy et al., 2017 to make the 19/35 nt RNA marker). The solutions were cooled for 2 min on ice and added to 1 μl Rnl2(1-249) K227Q ligase (NEB, Catalog no. M0351S) and incubated at 4°C overnight with shaking.

The next day the samples were added to 20 μl of formamide gel loading solution (50 mM EDTA, 0.05% (w/v) bromophenol blue and formamide at 100%) and the samples were loaded onto a 15% denaturing Urea-PAGE, the same was done with the 19/35 nt RNA marker, at both sides of the gel. After the run, the gel was wrapped in plastic film and exposed to a blank phosphorimager screen for at least 1 hour at −20°C. The markers and samples in between 19/35 nt RNA marker were cut from the gel. The recovered RNA was purified with phenol-chloroform extraction and ethanol precipitation. The RNA pellet was resuspended in 9 μl of water.

##### E. 5’ adapter ligation and cDNA synthesis

For the 5’ adapter ligation, the recovered RNA from last step (samples and RNA marker, in separate tubes) were mixed to 2 μl RNA ligase buffer containing ATP (500 mM Tris-HCl, pH 7.6, 100 mM MgCl_2_, 100 mM DTT and 10 mM ATP), 6 μl 50% PEG-8000 and 1 μl of 100 μM 5’ adapter. The resultant solutions were boiled for 1 min at 90°C, cooled in ice for 2 min and added to 2 μl of Rnl1 (NEB, Catalog no. M0204S). The reactions were incubated at 37°C for 1 hour with shaking. The samples were added to formamide gel loading solution, loaded onto a 12% denaturing Urea-PAGE and the RNA was recovered as described in item D after 3’ adapter ligation.

The ligated RNA was reverse transcribed with SuperScript^™^ III Reverse Transcriptase (Invitrogen^™^, Catalog no. 18080044), using the 3’ RT primer according to the manufacturer’s protocol.

##### F. PCR, purification, size selection and sequencing

The cDNA was amplified with Platinum Taq DNA polymerase (Thermo Fisher, Catalog no. 10966018) using 0.5 μM of a barcoded 3’ PCR-primer and 5’ PCR primer in a 100 μL reaction volume. The following protocol was used: 94°C, 2 min; 94°C, 30s; 60°C, 30s; 72°C, 15s for a total of 30 cycles. Starting from cycle 8, 10 μL of reaction mix were removed every 3 cycles and analyzed in a 2.5% agarose gel. The lowest number of cycles resulting in visible PCR product was chosen and a new 100 μl reaction volume PCR was set up. The result PCR was purified and concentrated using DNA Clean & Concentrator^™^-5 (Zymo Research, Catalog no. D4013).

Products of a size around 160 bp were isolated using a 3% agarose PippinPrep cassette (Sage Science, Catalog no. CSD3010) on a BluePippin device. After that, if the samples still presented a contaminant band below the expected size (linker-linker byproduct), a new round of PCR and size selection was made. The samples were quantified by TapeStation and sequenced on an Illumina HiSeq 3000 machine as single reads with 50 cycles. Analysis was performed as described previously using PARalyzer^34^ built into the PARpipe pipeline^34^ pipeline mapping the reads to human genome hg19.

Adapters and primers sequences used for PAR-CLIP are listed in Table S3.

#### Microscopy and image analysis

Immunostained cells were imaged on Delta Vision Core system (Applied Precision/GE Healthcare, Issaquah, WA) consisting of Olympus IX70 inverted microscope (Olympus America, Inc. Melville, NY) with 100X NA 1.4 oil immersion objective and a CoolSnap HQ 12-bit camera (Photometrics, Tucson, AZ) controlled by softWoRx 7.2.1 software. Filters used for imaging were FITC (Ex490/20; Em 528/38), RD-TR (Ex555/28; Em 617/73) and DAPI (Ex360/40; Em 457/50) of the 86000 Sedat Quadruple Filter Set (Chroma Technology Corp, Bellows Falls, VT). Z-stacks were acquired with the thickness interval of 10 μm each. Length of mitotic spindles were analyzed by measuring distance between two poles in a mitotic spindle and length across cellular cortex to cortex aligned with the spindle poles using “measure distance” tool in softWoRx. To prepare the figures, images were deconvolved, unless otherwise mentioned, with softWoRx and scaled manually to 8-bit using linear LUT and the same range of scaling for all the images.

#### Alternative splicing analysis

Alternative splicing analysis was done using fastq files from RNA-Seq made by poly (A) capture using rMATs.^70^ The splicing events were filtered for the ones that appeared in at least 5 reads, in which the PSI were ≥0.1 or ≤ −0.1 and FDR (False Discovery Rate) < 0.05 by using maser package (version 1.8.0) (http://www.bioconductor.org/packages/release/bioc/html/maser.html) for R (version 4.0.4) (https://www.R-project.org/).

#### K-mer or motif analysis

We used an in-house script to make the K-mer or motif analysis. It was implemented by counting all 5-mer occurrences within the binding site sequences and comparing these values with 5-mer abundances within a background file. The background file was built by matching each binding site with random continuous sequences of equal length taken from the region of the gene where that binding site was found, like intron, 3’UTR, CDS, etc.

#### Average coverage of binding sites

The visualization of binding site coverage (Figure 1H) was performed using the RCAS tool (version 1.12.0)^62^ using R software (version 4.0.4).

### QUANTIFICATION AND STATISTICAL ANALYSIS

The statistical analysis for all data was performed using at least three replicates. The significance of statical analyses were tested using two-tailed Student’s t-test when comparing two groups or one-way ANOVA when comparing more than four groups. Two-way ANOVA was performed for proliferation assays. Spearman’s coefficient correlation was used for correlation analysis between different combinations of PAR-CLIP replicates samples (Scatterplots from T to C conversions). Two-tailed Kolmogorov-Smirnov test was used for comparisons of cumulative distribution. Data were considered significant when P < 0.05. Prism software (version 9) was used to make the analysis. Details of experiments can be found in figure legends.

## Supplementary Material

1

2

3

4

5

## Figures and Tables

**Table:** KEY RESOURCES TABLE

REAGENT or RESOURCE	SOURCE	IDENTIFIER
Antibodies		
GAPDH rabbit monoclonal	Cell Signaling Technology	Cat#5174S
CDC14B rabbit polyclonal	Thermo Fischer Scientific	Cat#34-8900
Matrin3 rabbit polyclonal	Bethyl Laboratories^®^	Cat#A300-591A
IgG mouse	Santa Cruz Biotechnology	Cat#sc-2025; RRID: AB_737182
CENP-A mouse monoclonal	Abcam	Cat#ab13939; RRID: AB_300766
NUF2 rabbit monoclonal	Abcam	Cat#ab176556
EB1 mouse monoclonal	BD Biosciences	Cat#610534; RRID: AB_397891
UPF1 goat polyclonal	Bethyl Laboratories^®^	Cat#A300-038A
alpha tubulin mouse monoclonal	Abcam	Cat#ab7291; RRID: AB_2241126
gamma tubulin rabbit polyclonal	Abcam	Cat#ab11317; RRID: AB_297921
goat anti-rabbit DY 488	Thermo Fischer Scientific	Cat#35552
goat anti-rabbit DY 594	Thermo Fischer Scientific	Cat#35560
goat anti-mouse DY 488	Thermo Fischer Scientific	Cat#35502
goat anti-mouse DY 594	Thermo Fischer Scientific	Cat#35510
Bacterial and Virus Strains		
Subcloning Efficiency^™^ DH5α Competent Cells	Invitrogen^™^	Cat#18265017
Chemicals, Peptides, and Recombinant Proteins		
Opti-MEM^™^	Gibco	Cat#31985062
4-Thiouridine	Sigma-Aldrich	Cat#T4509
iScript^™^ cDNA Synthesis Kit	Biorad	Cat#1708890
FastStart Universal SYBR Green Master (Rox)	Millipore Sigma	Cat#4913914001
Pierce^™^ Protein A/G Magnetic Beads	Thermo Fisher Scientific	Cat#88803
Lipofectamine^™^ RNAiMAX Transfection Reagent	Invitrogen^™^	Cat#13778075
TRIzol^™^ Reagent	Invitrogen^™^	Cat#15596018
cOmplete^™^, Mini, EDTA-free Protease Inhibitor Cocktail	Millipore Sigma	Cat#11836170001
RNase T1	Thermo Fisher Scientific	Cat#EN0541
Alkaline Phosphatase, Calf intestinal (CIP)	NEB	Cat#M0290S
Rnl2(1-249) K227Q	NEB	Cat#M0351S
T4 PNK kinase	NEB	Cat#M0201S
Proteinase K	Roche	Cat#03450376103
Rnl1 ligase	NEB	Cat#M0204S
SuperScript^™^ III Reverse Transcriptase	Invitrogen^™^	Cat#18080044
Platinum Taq DNA polymerase	Thermo Fisher Scientific	Cat#10966018
Phusion^®^ High-Fidelity PCR Master Mix with HF Buffer	NEB	Cat#M0531S
MG132	Sigma-Aldrich	Cat#474790-10MG
ProLong^™^ Gold Antifade Mounting	Thermo Fisher Scientific	Cat#P36935
RIPA buffer	Life Technologies	Cat#89901
Monastrol	TOCRIS	Cat#1305
Critical Commercial Assays		
RNeasy Plus Mini Kit	Qiagen	Cat#74134
Illumina TruSeq Stranded mRNA Library Prep	Illumina	Cat#20020594
NEBNext rRNA Depletion Kit	NEB	Cat#E6318
NEBNext Ultra Directional RNA Library Prep Kit	NEB	Cat#E7760
Cell counting Kit 8	Dojindo Molecular Technologies, Inc.	Cat#CK04-13
3% PippinPrep	Sage Science	Cat#CSD3010
DNA Clean & Concentrator^™^-5	Zymo Research	Cat#D4013
Deposited Data		
Superseries of Raw and analyzed data from Ribo-depleted RNA-seq, Poly A capture RNA-seq, Iso-Seq and PAR-CLIP from this work	This paper	GEO: GSE203521
Ribo-depleted RNA-seq data from HCT116 cells after knockdown of Matrin3 using siRNAs	This paper	GEO: GSE203518
Poly A capture RNA-seq data from HCT116 cells after knockdown of Matrin3 using siRNAs	This paper	GEO: GSE203519
Iso-Seq data from HCT116 cells (no treatment)	This paper	GEO: GSE205565
PAR-CLIP data from Matrin3 in HCT116 cells using Matrin3 rabbit polyclonal antibody	This paper	GEO: GSE203520
Experimental Models: Cell Lines		
HCT116	ATCC	ATCC CCL-247
293T	ATCC	ATCC CRL-3216
HCT116- pCW-Cas9-Blast_empty vector	This paper	N/A
HCT116- pCW-Cas9-Blast_CDC14B-FLAG	This paper	N/A
HCEC-1CT	Evercyte	CHT-039-0229
IMR90	ATCC	ATCC CCL-186
WI38	ATCC	ATCC CCL-75
LOVO	ATCC	ATCC CCL-229
LS174T	ATCC	ATCC CL-188
LS180	ATCC	ATCC CL-187
Oligonucleotides		
Primers, see Table S3	This paper	N/A
ON-TARGETplus Human MATR3 (7157) siRNA - SMARTpool	Horizon Discovery	Cat# L-017382-00-0005
ON-TARGETplus Human UPF1 (5976) siRNA - SMARTpool	Horizon Discovery	Cat#L-011763-00-0005
Allstars Negative Control	Qiagen	Cat#1027281
siCDC14B-s_Sense: 5’ rGrCrUrArUrUrCrCrUrCrUrCrArCrArGrUrArArUrUrCrUTC 3’	IDT	N/A
siCDC14B-s_Antisense: 5’ rGrArArGrArArUrUrArCrUrGrUrGrArGrArGrGrArArUrArGrCrGrU 3’	IDT	N/A
Adapters and primers for PAR-CLIP	Benhalevy et al.^61^	See Table S3 for PAR-CLIP adapter and primer sequences
Recombinant DNA		
pCW-Cas9-Blast (modified empty vector)	gift from Mohan Babu (Addgene plasmid # 83481; http://n2t.net/addgene:83481; RRID:Addgene_83481)	N/A
pCW-Cas9-Blast-CDC14B-FLAG	This paper/gift from Mohan Babu (Addgene plasmid # 83481; http://n2t.net/addgene:83481; RRID:Addgene_83481)	N/A
Software and Algorithms		
RCAS version 1.12.0	Uyar et al.^62^	https://www.bioconductor.org/packages/release/bioc/html/RCAS.html
TopHat2 version 2.1.1	Kim et al.^63^	https://ccb.jhu.edu/software/tophat/index.shtml
STAR version 2.5.4a	Dobin et al.^64^	https://github.com/alexdobin/STAR
RSEM version 1.2.31	Li and Dewey^65^	https://deweylab.github.io/RSEM/
DESeq2 version 1.26.0	Love et al.^66^	https://bioconductor.org/packages/release/bioc/html/DESeq2.html
Trimmomatic version 0.36	Bolger et al.^67^	http://www.usadellab.org/cms/?page=trimmomatic
Trim Galore version 0.4.5	N/A	https://www.bioinformatics.babraham.ac.uk/projects/trim_galore/
Salmon version 0.14.1	Patro et al.^68^	https://github.com/COMBINE-lab/salmon
Sleuth version 0.30.0	Pimentel et al.^69^	https://github.com/pachterlab/sleuth
Wasabi version 1.0.1	N/A	https://github.com/COMBINE-lab/wasabi
PARalyzer version 1.5	Corcoran et al.^34^	https://ohlerlab.mdc-berlin.de/software/PARalyzer_85/
PARpipe	Corcoran et al.^34^	https://ohlerlab.mdc-berlin.de/software/PARpipe_119/
rMATS version 4.0.2	Shen et al.^70^	http://rnaseq-mats.sourceforge.net/
maser version 1.8.0	N/A	https://www.bioconductor.org/packages/release/bioc/html/maser.html
softWoRx 7.2.1	Cytiva	https://download.cytivalifesciences.com/cellanalysis/download_data/softWoRx/7.2.1/SoftWoRx.htm
SMRTlink (smrtlink-release_9.0.0.92188)	PacBio	https://www.pacb.com/support/software-downloads/
PacBio IsoSeq v3	PacBio	https://github.com/PacificBiosciences/IsoSeq
Minimap2	Li^71^	https://github.com/lh3/minimap2
SQANTI3	Tardaguila et al.^72^	https://github.com/ConesaLab/SQANTI3
R version 4.0.4	N/A	https://www.r-project.org/
Image J version 2.0.0-rc-43/1.52n	N/A	https://imagej.nih.gov/ij/
Imaris version 9.5.0	N/A	https://imaris.oxinst.com/
Prism version 9	Graph Pad Software	https://www.graphpad.com/scientific-software/prism/

## Highlights

- Matrin3 loss reduces cancer cell proliferation
- Matrin3 promotes proper target mRNA processing
- Matrin3 loss results in decreased CDC14B mRNA expression
- Matrin3-dependent CDC14B reduction results in abnormal mitotic spindle dynamics
