# Supplementary material for: Matrin3 regulates mitotic spindle dynamics by controlling alternative splicing of CDC14B
Source: Cell Rep. Author manuscript; Available in PMC 2023 Apr 26. (PMC10132239; doi:10.1016/j.celrep.2023.112260)
Supplement: 1 [file NIHMS1887310-supplement-1.pdf]

Cell Reports, Volume *42*

Supplemental information

Matrin3 regulates mitotic spindle dynamics

by controlling alternative splicing of CDC14B

Bruna R. Muys, Roshan L. Shrestha, Dimitrios G. Anastasakis, Lorinc Pongor, Xiao Ling Li, Ioannis Grammatikakis, Ahsan Polash, Raj Chari, Myriam Gorospe, Curtis C. Harris, Mirit I. Aladjem, Munira A. Basrai, Markus Hafner, and Ashish Lal

Supplemental Information

Figure S1

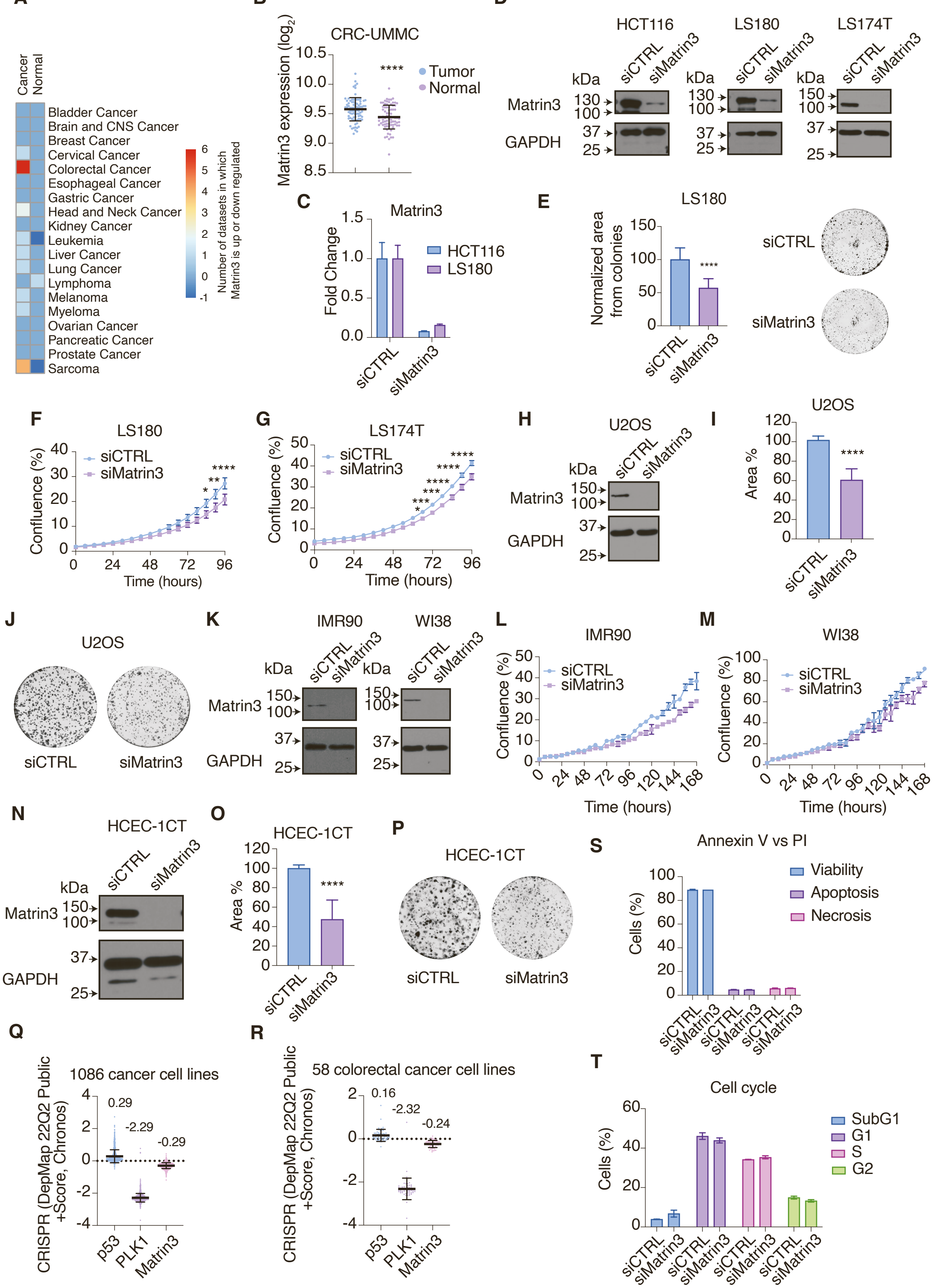
A
Cancer
Normal
Bladder Cancer
Brain and CNS Cancer
Breast Cancer
Cervical Cancer
Colorectal Cancer
Esophageal Cancer
Gastric Cancer
Head and Neck Cancer
Kidney Cancer
Leukemia
Liver Cancer
Lung Cancer
Lymphoma
Melanoma
Myeloma
Ovarian Cancer
Pancreatic Cancer
Prostate Cancer
Sarcoma
Number of datasets in which Matrin3 is up or down regulated
B
CRC-UMMC
Matrin3 expression (log2)
Tumor
Normal
****
C
Matrin3
Fold Change
HCT116
LS180
siCTRL
siMatrin3
D
HCT116
LS180
LS174T
kDa
Matrin3
GAPDH
siCTRL
siMatrin3
E
LS180
Normalized area from colonies
siCTRL
siMatrin3
****
F
LS180
Confluence (%)
Time (hours)
siCTRL
siMatrin3
G
LS174T
Confluence (%)
Time (hours)
siCTRL
siMatrin3
H
U2OS
kDa
Matrin3
GAPDH
siCTRL
siMatrin3
I
U2OS
Area %
siCTRL
siMatrin3
****
J
U2OS
siCTRL
siMatrin3
K
IMR90
WI38
kDa
Matrin3
GAPDH
siCTRL
siMatrin3
L
IMR90
Confluence (%)
Time (hours)
siCTRL
siMatrin3
M
WI38
Confluence (%)
Time (hours)
siCTRL
siMatrin3
N
HCEC-1CT
kDa
Matrin3
GAPDH
siCTRL
siMatrin3
O
HCEC-1CT
Area %
siCTRL
siMatrin3
****
P
HCEC-1CT
siCTRL
siMatrin3
S
Annexin V vs PI
Cells (%)
Viability
Apoptosis
Necrosis
siCTRL
siMatrin3
Q
1086 cancer cell lines
CRISPR (DepMap 22Q2 Public +Score, Chronos)
0.29
-2.29
-0.29
p53
PLK1
Matrin3
R
58 colorectal cancer cell lines
CRISPR (DepMap 22Q2 Public +Score, Chronos)
0.16
-2.32
-0.24
p53
PLK1
Matrin3
T
Cell cycle
Cells (%)
SubG1
G1
S
G2
siCTRL
siMatrin3

Figure S1. Matrin3 is upregulated in CRC and its knockdown leads to growth defects in multiple cell types. Related to Figure 1.

A. Heatmap of Matrin3 mRNA expression changes in different tumors compared to normal tissue in the Oncomine datasets. Threshold was set to ≥ 2-fold expression change and p <0.0001. Matrin3 mRNA is overexpressed mostly in colorectal cancer compared to other cancer types. **B.** Matrin3 mRNA expression in tumors derived from colorectal cancer the from University of Maryland Medical Center (UMMC) cohort compared to respective normal samples. Unpaired, two-sided t-test; N Tumor = 83 and N Normal = 79. Error bars = SD and ****p<0.0001. **C.** RT-qPCR quantification of Matrin3 knockdown using siRNAs against Matrin3 in HCT116 and LS180 cells. N = 2. Error bars = SD. **D.** Immunoblots for GAPDH and Matrin3 from HCT116, LS180 and LS174T whole cell lysates following Matrin3 knockdown. **E.** Colony formation assays after Matrin3 knockdown in LS180 cells. Unpaired, two-sided t-test; N = 3. Error bars = SD and ****p<0.0001. **F and G.** Cell proliferation assays using Incucyte® after Matrin3 knockdown in LS180 and LS174T cells, respectively. Two-way ANOVA test; N = 3; Error bars = SEM, *p<0.05, **p<0.01, ***p<0.001 and ****p<0.0001. **H.** Immunoblots for GAPDH and Matrin3 from U2OS whole cell lysate following Matrin3 knockdown. **I.** Colony formation assays from U2OS cells after Matrin3 knockdown. Unpaired, two-sided t-test; N = 3; Error bars = SD and ****p<0.0001. **J.** Representative images of U2OS colonies for the data in panel I. **K.** Immunoblots for GAPDH and Matrin3 from IMR90 and WI38 whole cell lysates following Matrin3 knockdown. **L and M.** Representative image of cell proliferation assay using Incucyte® after Matrin3 knockdown in IMR90 (B) and WI38 (C) cells, respectively. Representative image from one of 3 biological experiments is shown. Error bars = SEM. **N.** Immunoblots for GAPDH and Matrin3 from HCEC-1CT whole cell lysates following Matrin3 knockdown. **O.** Colony formation assays from HCEC-

1-CT cells after Matrin3 knockdown. Unpaired, two-sided t-test; N = 3; Error bars = SD and ****p<0.0001. **P.** Representative images of HCEC-1CT colonies for the data in panel O. **Q and R**. Graphs showing DepMap scores for Matrin3, p53 (positive control for a growth suppressive gene) and PLK1 (positive control for a growth promoting gene) in diverse cancer cell lines (Q) and CRC cell lines only in (R). Error bars = SD. **S and T.** The effect of Matrin3 knockdown on apoptosis and cell cycle was determined by Annexin V staining and/or PI staining and FACS analysis. Representative image from one of 2 biological experiments is shown. Error bars = SD.

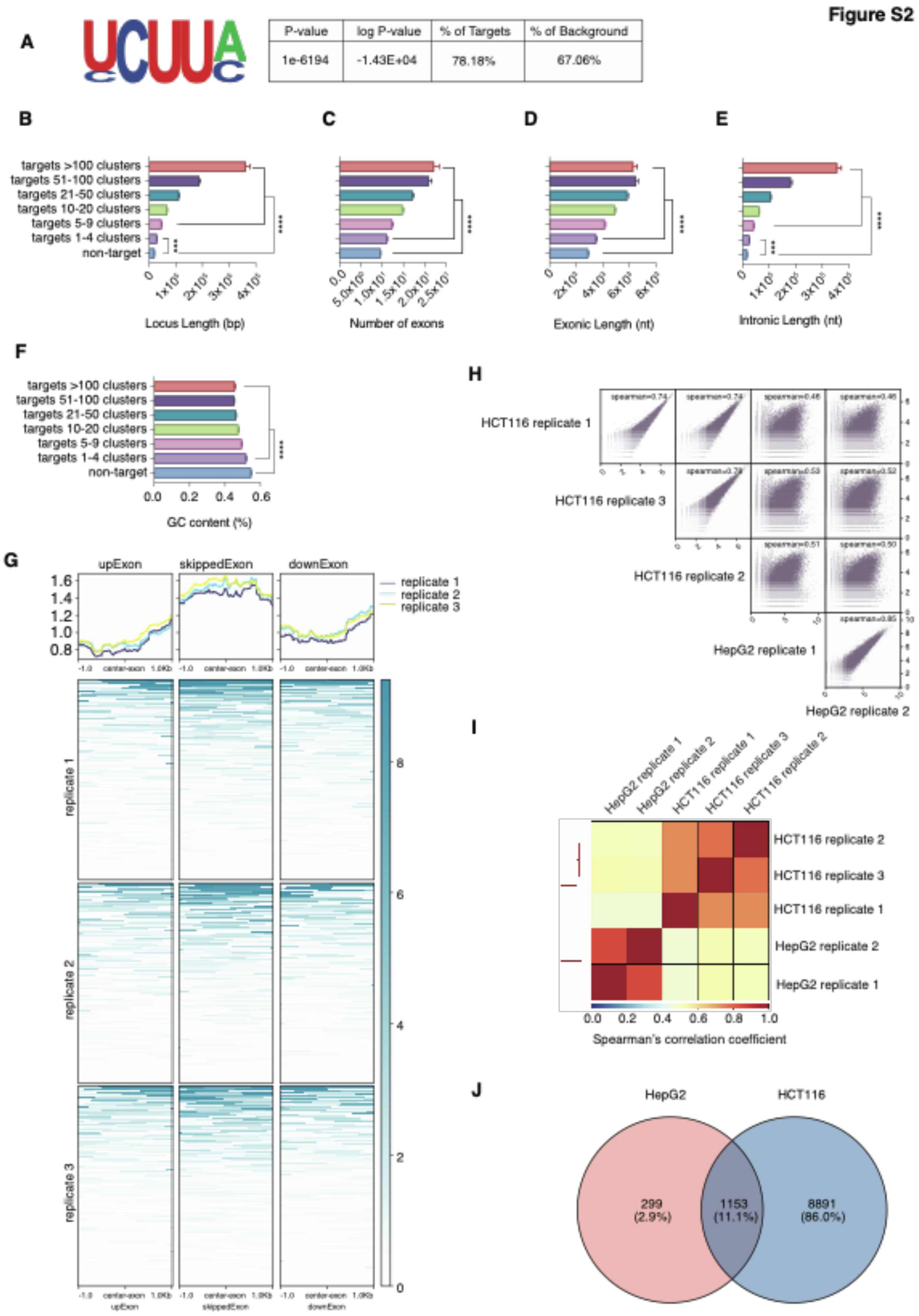
Figure S2
A
P-value | log P-value | % of Targets | % of Background
1e-6194 | -1.43E+04 | 78.18% | 67.06%
B
C
D
E
targets >100 clusters
targets 51-100 clusters
targets 21-50 clusters
targets 10-20 clusters
targets 5-9 clusters
targets 1-4 clusters
non-target
Locus Length (bp)
Number of exons
Exonic Length (nt)
Intronic Length (nt)
F
GC content (%)
G
upExon
skippedExon
downExon
replicate 1
replicate 2
replicate 3
center-exon
H
HCT116 replicate 1
HCT116 replicate 3
HCT116 replicate 2
HepG2 replicate 1
HepG2 replicate 2
I
Spearman's correlation coefficient
J
HepG2
HCT116
299 (2.9%)
1153 (11.1%)
8891 (86.0%)

Figure S2. Characterization of Matrin3 binding on its target RNAs. Related to Figure 1.

A. Top Matrin3-binding motif derived from combined Matrin3 PAR-CLIP replicates and its statistic data. **B-F:** Gene architecture of Matrin3 targets. Matrin3 PAR-CLIP targets with different binding site numbers were binned according to: **B.** Locus length in base pairs. One-way ANOVA test, ***p<0.001 and ****p<0.0001. **C.** Number of exons. One-way ANOVA test and ****p<0.0001. **D.** Exonic length in nucleotide number (nt). One-way ANOVA test and ****p<0.0001. **E.** Intronic length in nucleotide number (nt). One-way ANOVA test, ***p<0.001 and ****p<0.0001. **F.** GC content in percentage (%). One-way ANOVA test and ****p<0.0001. Non-targets, N = 2637; Targets with: 1-4 clusters N = 1183; 5-9 clusters N = 1185; 10-20 clusters N = 1122; 21-50 clusters N = 1007; 51-100 clusters N = 399; >100 clusters N = 167; Error bars = SEM. **G**. Top: metagene graph showing the coverage of reads from Matrin3 PAR-CLIP replicates in upstream, included or skipped, and downstream exons derived from our splicing analysis. Bottom: Same as in top part but showing the individual reads per replicate. **H**. Scatter plots from overlap among binding sites of Matrin3 defined by genomic coordinates. **I.** Heatmap of Spearman's correlation coefficient from overlap among binding sites of Matrin3 defined by genomic coordinates. **J.** Comparison among genes containing binding sites in introns in HepG2 and HCT116 cell lines.

Figure S3

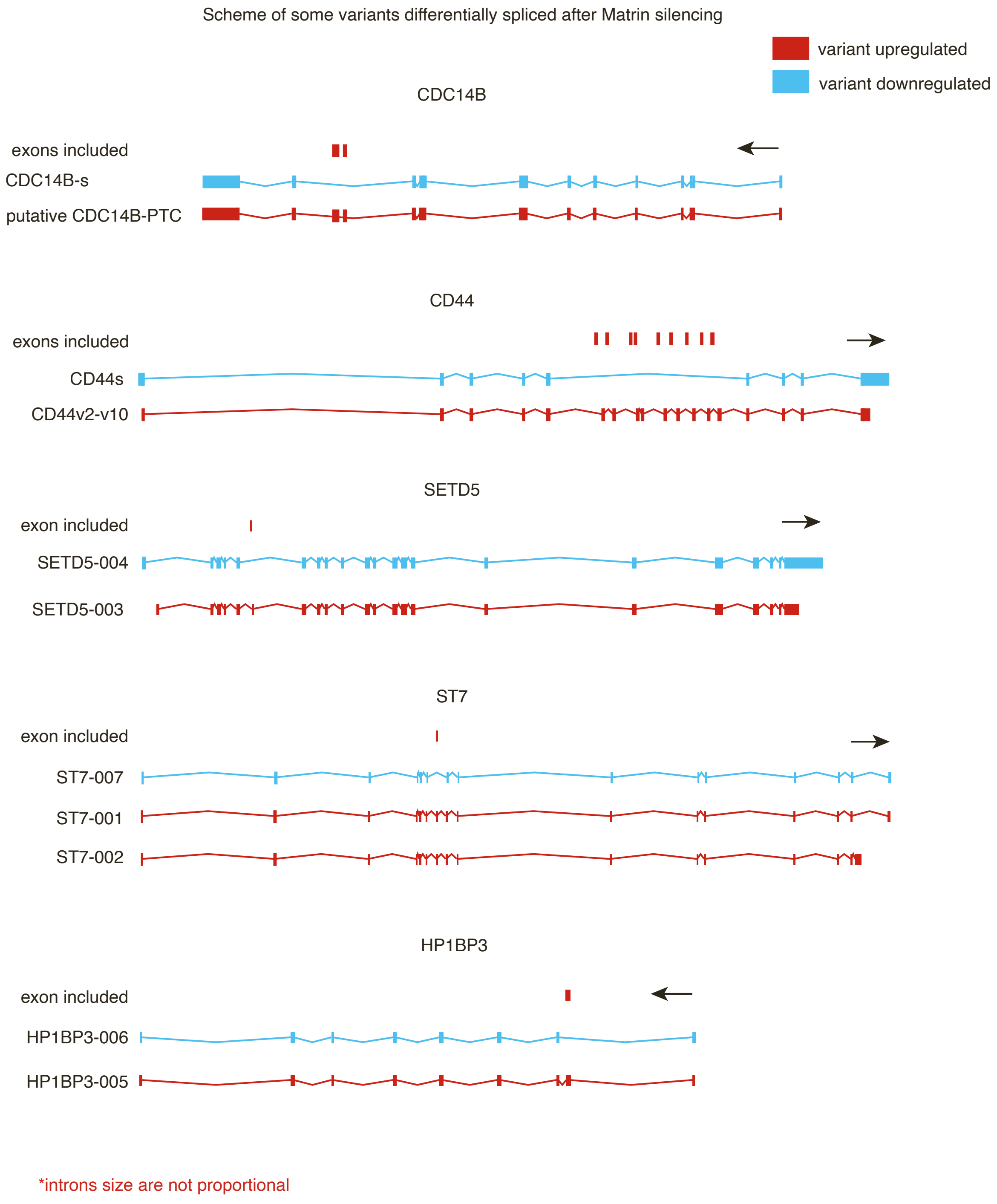
Scheme of some variants differentially spliced after Matrin silencing
variant upregulated
variant downregulated
CDC14B
exons included
CDC14B-s
putative CDC14B-PTC
CD44
exons included
CD44s
CD44v2-v10
SETD5
exon included
SETD5-004
SETD5-003
ST7
exon included
ST7-007
ST7-001
ST7-002
HP1BP3
exon included
HP1BP3-006
HP1BP3-005
*introns size are not proportional

54

Figure S3. Schematic representations of variants with altered expression after Matrin3 knockdown from Figures 2C and 2D. Related to Figure 2.

The image shows the exon(s) that was (were) included. The introns are not proportional to the size of the pre-mRNAs.

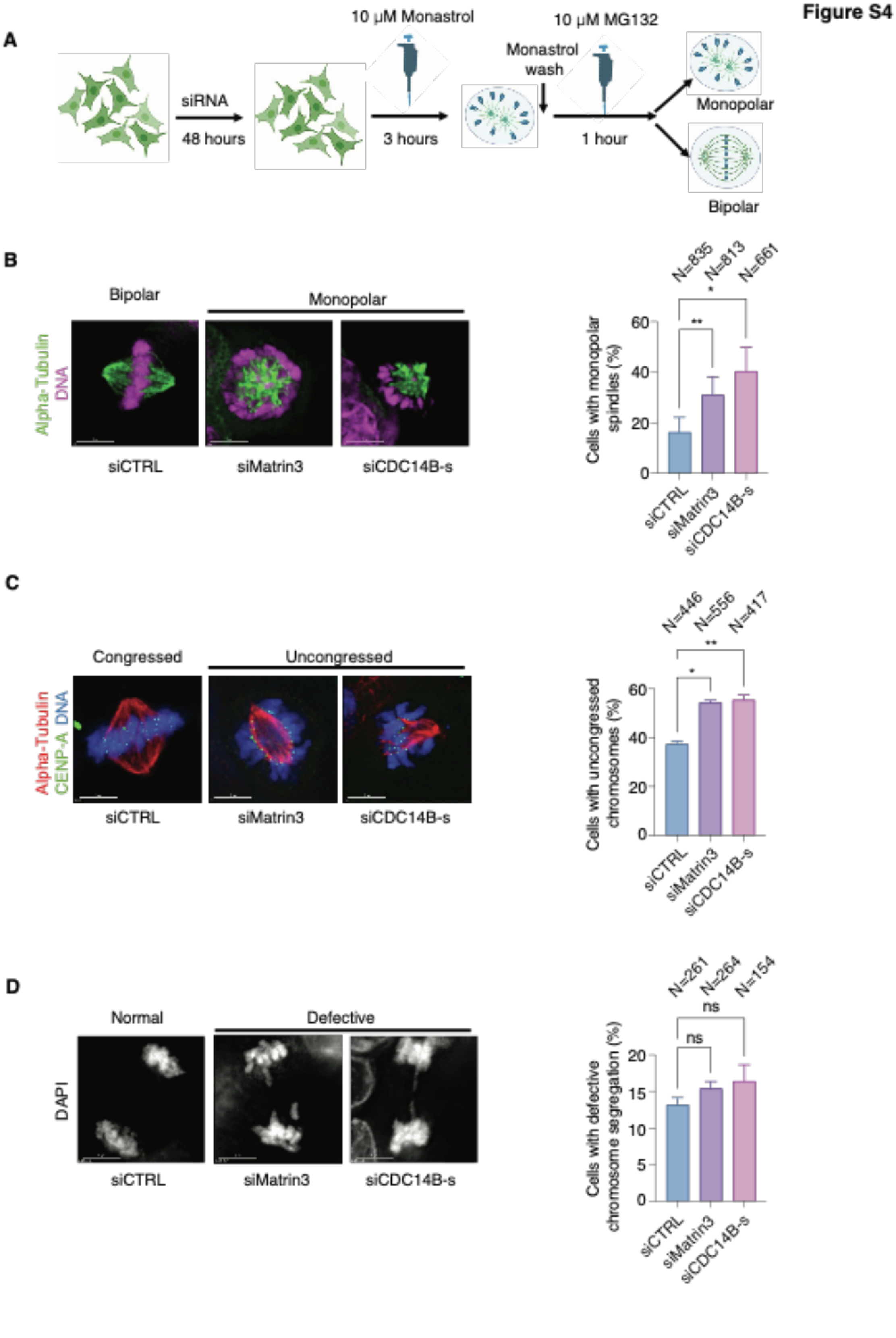
Figure S4
A
10 µM Monastrol
10 µM MG132
Monastrol wash
siRNA
48 hours
3 hours
1 hour
Monopolar
Bipolar
B
Bipolar
Monopolar
Alpha-Tubulin DNA
siCTRL
siMatrin3
siCDC14B-s
N=835
N=813
N=661
Cells with monopolar spindles (%)
C
Congressed
Uncongressed
Alpha-Tubulin CENP-A DNA
N=446
N=556
N=417
Cells with uncongressed chromosomes (%)
D
Normal
Defective
DAPI
N=261
N=264
N=154
ns
Cells with defective chromosome segregation (%)

60

Figure S4. Effect of Matrin3 knockdown on spindle polarity and chromosome congression. Related to Figure 4.

A. Scheme of monastrol wash out assay for mitotic spindle bipolarity. **B.** Representative immunofluorescence images of monastrol washed off mitotic cells fixed following MG132 treatment, and immunostained with alpha-tubulin for spindle microtubules after treatment with Matrin3, CDC14B-s or control siRNAs. Chromosomes were stained with DAPI, Scale bar: 5 μm. (Right panel) Bar graph of proportion of cells with monopolar spindles following monastrol wash out as illustrated in A. "N" denotes number of cells analyzed. Unpaired, two-sided t-test; Error bars = SD, *p<0.05 and **p<0.01. **C.** Representative immunofluorescence images of asynchronous cells in mitosis and immunostained with alpha-tubulin for spindle microtubules and CENP-A for centromeres after treatment with Matrin3, CDC14B-s or control siRNAs. Chromosomes were stained with DAPI, Scale bar: 5 μm. (Right panel). Bar graph of proportion of cells with uncongressed chromosomes. "N" denotes number of cells analyzed. Unpaired, two-sided t-test; Error bars = SD, *p<0.05 and **p<0.01. **D.** Representative immunofluorescence images of asynchronous cells in anaphase and stained with DAPI chromosomes after treatment with Matrin3, CDC14B-s or control siRNAs. Scale bar: 5 μm. (Right panel). Bar graph of proportion of cells with defective chromosomes segregation. "N" denotes number of cells analyzed. Unpaired, two-sided t-test; Error bars = SD, ns = non-significant.
